# Supplementary material for: Trends of long-term opioid therapy and subsequent discontinuation among people with chronic non-cancer pain in UK primary care: A retrospective cohort study
Source: PLoS One. 2025 Jun 26;20(6):e0326604. doi: 10.1371/journal.pone.0326604 (PMC12200650; doi:10.1371/journal.pone.0326604)
Supplement: S2 Fig — (DOCX) [file pone.0326604.s002.docx]

# **S2 Fig. Definition of L-TOT users and L-TOT discontinuers**


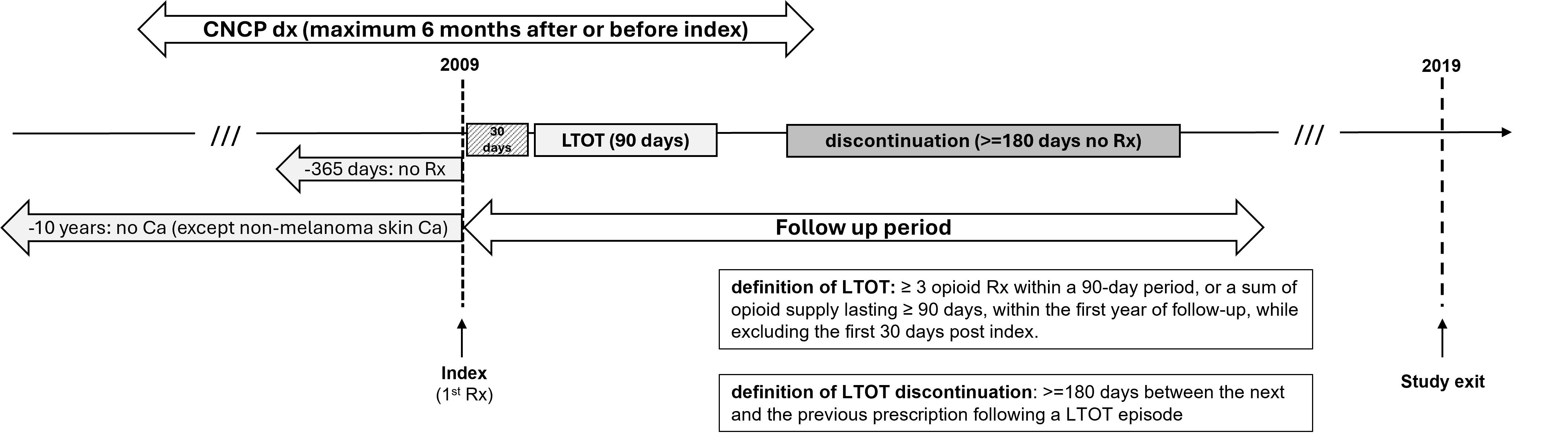


Note: dx=diagnosis; Rx=description; Ca=cancer
