# Supplementary material for: Trends of long-term opioid therapy and subsequent discontinuation among people with chronic non-cancer pain in UK primary care: A retrospective cohort study
Source: PLoS One. 2025 Jun 26;20(6):e0326604. doi: 10.1371/journal.pone.0326604 (PMC12200650; doi:10.1371/journal.pone.0326604)
Supplement: S3 Fig — (DOCX) [file pone.0326604.s003.docx]

# **S3 Fig. Decisions made for opioid drug preparation to derive daily dose**


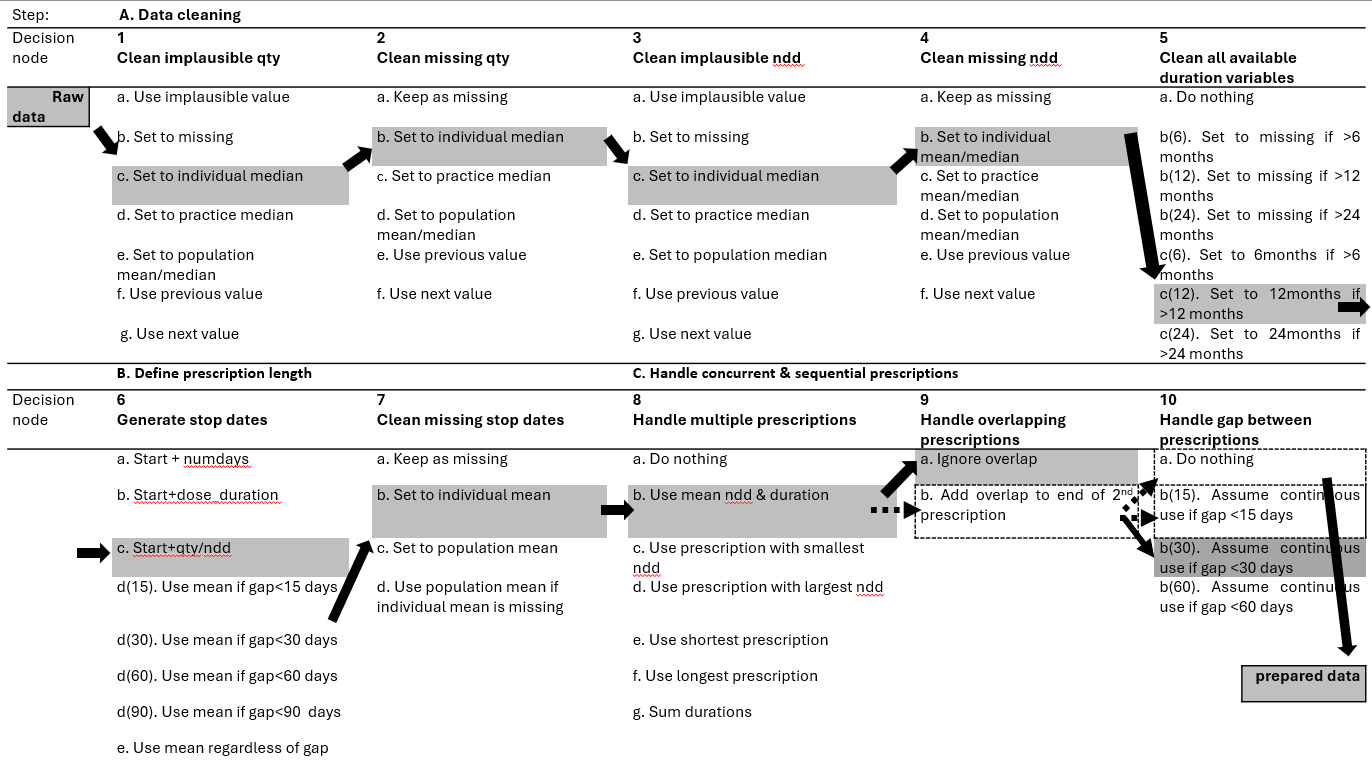


Note: qty=quantity; ndd=daily dose; numdays=number of days; The decisions in the dark grey boxes were made for the primary analysis, while those in the dashed boxes were used for the sensitivity analyses.
