# Supplementary material for: Trends of long-term opioid therapy and subsequent discontinuation among people with chronic non-cancer pain in UK primary care: A retrospective cohort study
Source: PLoS One. 2025 Jun 26;20(6):e0326604. doi: 10.1371/journal.pone.0326604 (PMC12200650; doi:10.1371/journal.pone.0326604)
Supplement: S1 Table — (DOCX) [file pone.0326604.s004.docx]

# **S1 Table. CNCP code list**

| **medcodeid** | **Description** |
| --- | --- |
| 1634018 | Occipital headache |
| 8258016 | Repetitive strain injury |
| 10927012 | Congenital fusion of sacroiliac joint |
| 16579010 | Meningococcal arthropathy |
| 16833013 | Ankylosing spondylitis |
| 30355011 | Post-herpetic trigeminal neuralgia |
| 41990019 | Headache |
| 58058010 | Coccygodynia |
| 58293010 | Pyogenic arthritis of multiple sites |
| 64723010 | Tuberculosis of spinal meninges |
| 65676015 | Congenital anomaly of spinal meninges |
| 68961012 | Thoracic spinal stenosis |
| 69073019 | Temporal headache |
| 71168014 | Peripheral neuropathy |
| 80709010 | Gouty arthritis |
| 82623012 | Ophthalmoplegic migraine |
| 89215016 | Gonococcal spondylitis |
| 98501012 | Hemiplegic migraine |
| 99770017 | Sprain of cruciate ligament of knee |
| 108529013 | Polymyalgia rheumatica |
| 118420016 | Salmonella arthritis |
| 126406018 | Spinal stenosis |
| 127546017 | Epidemic cervical myalgia |
| 158460019 | Ophthalmic migraine |
| 178449010 | Ankylosis of joint |
| 178460018 | Periarthritis of wrist |
| 251645017 | H/O: migraine |
| 251646016 | H/O: trigeminal neuralgia |
| 251794010 | H/O: rheumatoid arthritis |
| 252316013 | C/O - upper back ache |
| 293392012 | Gouty arthropathy |
| 297456010 | Phantom limb syndrome |
| 297551015 | Chronic painful diabetic neuropathy |
| 297554011 | Polyneuropathy in amyloidosis |
| 297564019 | Polyneuropathy in porphyria |
| 297566017 | Polyneuropathy in sarcoidosis |
| 299135012 | Malleus ankylosis |
| 305093012 | Menopausal headache |
| 309459014 | Staphylococcal arthritis and polyarthritis |
| 309477016 | Sexually acquired reactive arthropathy of multiple sites |
| 309518017 | Arthropathy in Whipple's disease |
| 309555017 | Helminthiasis with arthropathy of the ankle and foot |
| 309556016 | Helminthiasis with arthropathy of multiple sites |
| 309570016 | Reactive arthropathy of hip |
| 309572012 | Reactive arthropathy of knee |
| 309574013 | Reactive arthropathy of ankle |
| 309575014 | Reactive arthropathy of subtalar joint |
| 309576010 | Reactive arthropathy of talonavicular joint |
| 309744019 | Arthropathy in Crohn's disease |
| 309749012 | Arthropathy associated with dermatological disorders |
| 309787016 | Rheumatoid arthritis of cervical spine |
| 309790010 | Rheumatoid arthritis of sternoclavicular joint |
| 309792019 | Rheumatoid arthritis of elbow |
| 309794018 | Rheumatoid arthritis of wrist |
| 309803012 | Rheumatoid arthritis of subtalar joint |
| 309816012 | Flare of rheumatoid arthritis |
| 309877012 | Erosive osteoarthrosis |
| 309878019 | Heberden's nodes with arthropathy |
| 309892013 | Localised, primary osteoarthritis of the hand |
| 309914014 | Localised, secondary osteoarthritis |
| 309933016 | Localised, secondary osteoarthritis of the ankle and foot |
| 310053015 | Traumatic arthropathy of the hand |
| 310054014 | Traumatic arthropathy of the pelvic region and thigh |
| 310062018 | Traumatic arthropathy-elbow |
| 310085017 | Allergic arthritis of the hand |
| 310086016 | Allergic arthritis of the pelvic region and thigh |
| 310088015 | Allergic arthritis of the ankle and foot |
| 310099018 | Climacteric arthritis of the hand |
| 310102018 | Climacteric arthritis of the ankle and foot |
| 310159018 | Generalised arthritis |
| 310543012 | Ankylosis of the wrist joint |
| 310544018 | Wrist joint ankylosis |
| 310551010 | Knee joint ankylosis |
| 310552015 | Ankle joint ankylosis |
| 310787017 | Palindromic rheumatism of multiple sites |
| 310818014 | Arthralgia of sternoclavicular joint |
| 310826018 | Arthralgia of wrist |
| 311112016 | Brucella spondylitis |
| 311114015 | Neuropathic spondylopathy |
| 311230016 | Idiopathic thoracic spinal stenosis |
| 311231017 | Degenerative thoracic spinal stenosis |
| 311238011 | Degenerative lumbar spinal stenosis |
| 311239015 | Iatrogenic lumbar spinal stenosis |
| 311252013 | Lumbago with sciatica |
| 311285017 | Thoracic spine ankylosis |
| 311287013 | Lumbar spine ankylosis |
| 311288015 | Atlanto-occipital instability |
| 311292010 | Thoracic spine instability |
| 311307012 | Rheumatism, excluding the back |
| 311687015 | Rheumatism and fibrositis unspecified |
| 311690014 | Muscular rheumatism |
| 312802011 | Thoracic spinal meningocele |
| 319764019 | Closed spinal dislocation with anterior thoracic cord lesion |
| 319765018 | Closed spinal dislocation with central thoracic cord lesion |
| 319770013 | Closed spinal dislocation with central lumbar cord lesion |
| 319771012 | Closed spinal dislocation with posterior lumbar cord lesion |
| 319836018 | Closed spinal subluxation with complete cervical cord lesion |
| 319838017 | Closed spinal subluxation with central cervical cord lesion |
| 319865012 | Closed spinal subluxation with complete thoracic cord lesion |
| 319871018 | Closed spinal subluxation with anterior lumbar cord lesion |
| 319872013 | Closed spinal subluxation with central lumbar cord lesion |
| 319873015 | Closed spinal subluxation with posterior lumbar cord lesion |
| 320119017 | Sprain, shoulder joint, posterior |
| 320122015 | Sprain, triceps tendon |
| 320137016 | Sprain, elbow joint, medial collateral ligament |
| 320139018 | Radiohumeral sprain |
| 320140016 | Ulnohumeral sprain |
| 320145014 | Sprain of wrist and hand |
| 320148011 | Carpal joint sprain |
| 320150015 | Distal radioulnar joint sprain |
| 320167010 | Sprain volar intercarpal ligament or V ligament |
| 320178019 | Metacarpophalangeal sprain |
| 320180013 | Midcarpal joint sprain |
| 320212019 | Sprain wrist flexors |
| 320218015 | Sprain, flexor digitorum profundus tendon |
| 320219011 | Sprain, extensor digitorum tendon |
| 320258018 | Sprain of ankle and foot |
| 320260016 | Sprain, ankle joint, medial |
| 320262012 | Sprain, ankle joint, lateral |
| 320282011 | Sprain pelvic ligament |
| 320284012 | Sprain, lumbosacral ligament |
| 320287017 | Sprain, sacrospinous ligament |
| 320289019 | Sprain, iliolumbar ligament |
| 320295018 | Neck sprain |
| 320472012 | Open division sacroiliac ligament |
| 320558017 | Chondrocostal joint sprain |
| 320563018 | Sternoclavicular sprain |
| 320564012 | Chondrosternal sprain |
| 320565013 | Xiphoid cartilage sprain |
| 320567017 | Pelvis sprain or complete tear |
| 323827010 | Open injury sciatic nerve |
| 325265011 | Dislocations, sprains and strains involving head with neck |
| 345367011 | Chronic post-traumatic headache |
| 345580013 | Intercostal neuropathy |
| 346003014 | Intracranial destruction of trigeminal ganglion |
| 354567013 | Beta-2 microglobulin arthropathy |
| 356490015 | Amyloid arthropathy |
| 356491016 | Arthropathy in amyloidosis |
| 359267014 | Wrist pyogenic arthritis |
| 359310019 | Juvenile arthritis in psoriasis |
| 359321011 | Distal interphalangeal psoriatic arthropathy |
| 359433019 | Osteoarthritis of lumbar spine |
| 359513016 | Giant cell arteritis with polymyalgia rheumatica |
| 359719015 | Viral myalgia |
| 360154013 | Postmeningococcal arthritis |
| 369417015 | Neuropathic pain |
| 397840012 | H/O: musculoskeletal disease |
| 397993014 | Frontal headache |
| 400175010 | Generalised osteoarthritis of the hand |
| 400224017 | Joint ankylosis of the pelvic region and thigh |
| 400226015 | Joint ankylosis of the ankle and foot |
| 400246013 | Arthralgia of shoulder |
| 400249018 | Arthralgia of the pelvic region and thigh |
| 402911018 | Sprain of elbow and forearm |
| 405550017 | Congenital instability of hip joint |
| 411067016 | FH: Arthritis |
| 411301012 | Shoulder strain |
| 416143014 | Lumbago |
| 423262010 | Open wound of sacroiliac region |
| 453181013 | Lumbosacral strain |
| 457118013 | Localised, primary osteoarthritis of the wrist |
| 457121010 | Localised, primary osteoarthritis of toe |
| 481611016 | Jaw sprain |
| 481614012 | Temporomandibular sprain |
| 481879010 | Hip joint ankylosis |
| 484889013 | Ankylosing vertebral hyperostosis |
| 489598015 | Lumbosacral instability |
| 493899010 | Ankle sprain |
| 496626018 | Arthropathy due to hypersensitivity reaction |
| 502155015 | Trigeminal (5th) nerve injury |
| 1219617015 | Arthropathy due to fungal infection |
| 1224865018 | Hereditary motor and sensory neuropathy type IV |
| 1229825015 | Pyogenic arthritis of the forearm |
| 1230691015 | Arthralgia of hip |
| 1232174014 | Pyogenic arthritis of the hand |
| 1235651015 | Familial amyloid polyneuropathy type III |
| 1494757018 | Thyroid cartilage sprain |
| 1494890011 | Sprain of hip and thigh |
| 1494891010 | Thigh sprain |
| 1777664015 | Hereditary motor and sensory neuropathy |
| 1777779015 | Amyloid polyneuropathy type I |
| 1785082014 | Rheumatic pain |
| 1786078018 | Tension headache |
| 1786163010 | Hereditary motor and sensory neuropathy type II |
| 2477015019 | Andrade type amyloid polyneuropathy |
| 2838283014 | Paroxysmal hemicrania |
| 230611000006118 | Phantom limb syndrome with pain |
| 303071000000118 | History of irritable bowel syndrome |
| 878961000006118 | Tuberculosis - meninges/CNS |
| 883161000006115 | Drug/toxic polyneuropathy |
| 886361000006114 | Irritable bowel - IBS |
| 889881000006119 | Osteoarthritis - elbow joint |
| 889901000006117 | Osteoarthritis - hand joint |
| 890371000006116 | Ankylosis - multiple joint |
| 890421000006111 | Ankylosis - hand joint |
| 890431000006114 | Ankylosis - hip joint |
| 890451000006119 | Ankylosis - ankle/foot |
| 890981000006117 | Spinal stenosis excl. cervical |
| 891861000006112 | Neuralgia/neuritis - NOS |
| 892321000006118 | Hypertroph. pulm. osteoarthrop |
| 895351000006119 | #Sacrum/coccyx + cord lesion |
| 896171000006115 | Dislocations/sprains/strains |
| 896231000006112 | Sprain - wrist |
| 896241000006119 | Sprained finger/thumb |
| 896261000006115 | Sprained thigh - upper leg |
| 896291000006111 | Sprained knee |
| 896301000006112 | Sprain - lateral knee ligament |
| 896321000006119 | Sprain -cruciate knee ligament |
| 896341000006114 | Sprained ankle |
| 896441000006118 | Sacral/coccyx sprain |
| 896481000006112 | Sprained ribs |
| 896491000006110 | Sprained sternum |
| 991391000006118 | Sprain - hand NOS |
| 991411000006118 | Sprain - ankle NOS |
| 991421000006114 | Sprain - foot NOS |
| 2214301000000116 | Chronic regional pain syndrome |
| 2254241000000117 | On musculoskeletal care pathway |
| 2360741000000115 | Axial spondyloarthritis |
| 2415191000000113 | Diabetic peripheral neuropathic pain |
| 309704014 | Crystal arthropathy NOS, of the pelvic region and thigh |
| 317148013 | [D]Nervous or musculoskeletal symptoms NOS |
| 41386013 | Fibromyalgia |
| 123542016 | Pauciarticular juvenile rheumatoid arthritis |
| 297355013 | Other forms of migraine NOS |
| 297428017 | Trigeminal neuralgia NOS |
| 297430015 | Trigeminal nerve disorder NOS |
| 297493016 | Other upper limb mononeuritis |
| 297511018 | Hereditary and idiopathic peripheral neuropathy |
| 297530018 | Other idiopathic peripheral neuropathy NOS |
| 299338017 | [X]Other migraine |
| 299361015 | [X]Other specified mononeuropathies |
| 299362010 | [X]Other mononeuropathies in diseases classified elsewhere |
| 299364011 | [X]Other hereditary and idiopathic neuropathies |
| 299366013 | [X]Other specified polyneuropathies |
| 308730016 | Psoriatic arthropathy NOS |
| 309441011 | Pyogenic arthritis of unspecified site |
| 309485013 | Arthropathy in Behcet's syndrome of the forearm |
| 309488010 | Arthropathy in Behcet's syndrome of the lower leg |
| 309492015 | Arthropathy in Behcet's syndrome NOS |
| 309548019 | Helminthiasis with arthropathy of unspecified site |
| 309554018 | Helminthiasis with arthropathy of the lower leg |
| 309557013 | Helminthiasis with arthropathy of other specified site |
| 309600012 | Infective arthritis NOS, of the pelvic region and thigh |
| 309601011 | Infective arthritis NOS, of the lower leg |
| 309605019 | Infective arthritis NOS, of acromioclavicular joint |
| 309606018 | Infective arthritis NOS, of elbow |
| 309614012 | Infective arthritis NOS, of hip |
| 309625015 | Infective arthritis NOS, of multiple sites |
| 309682019 | Gouty arthritis of other specified site |
| 309687013 | Other crystal arthropathies of the shoulder |
| 309689011 | Other crystal arthropathies of the forearm |
| 309690019 | Other crystal arthropathies of the hand |
| 309699018 | Crystal arthropathy NOS, site unspecified |
| 309760015 | Other general diseases with associated arthropathy |
| 309761016 | Arthritis associated with other disease, shoulder |
| 309766014 | Arthritis associated with other disease, wrist |
| 309778013 | Arthritis associated with other disease, other tarsal joint |
| 309783017 | Arthropathy associated with disorders EC NOS |
| 309911018 | Localised, primary osteoarthritis NOS |
| 309977011 | Oligoarticular osteoarthritis, unspecified, of hand |
| 310010015 | Osteoarthritis NOS, other specified site |
| 310014012 | Osteoarthritis NOS, of elbow |
| 310026019 | Osteoarthritis NOS, of ankle |
| 310029014 | Osteoarthritis NOS, of other tarsal joint |
| 310049017 | Traumatic arthropathy of unspecified site |
| 310052013 | Traumatic arthropathy of the forearm |
| 310084018 | Allergic arthritis of the forearm |
| 310087013 | Allergic arthritis of the lower leg |
| 310089011 | Allergic arthritis of other specified site |
| 310139017 | Transient arthropathy of other tarsal joint |
| 310152010 | Unspecified polyarthropathy of the hand |
| 310154011 | Unspecified polyarthropathy of the lower leg |
| 310157016 | Unspecified polyarthropathy of multiple sites |
| 310183014 | Other specified arthropathy of the shoulder region |
| 310184015 | Other specified arthropathy of the upper arm |
| 310187010 | Other specified arthropathy of the pelvic region and thigh |
| 310188017 | Other specified arthropathy of the lower leg |
| 310189013 | Other specified arthropathy of the ankle and foot |
| 310537010 | Joint ankylosis of other specified site |
| 310538017 | Ankylosis of other joint of the shoulder girdle |
| 310778014 | Palindromic rheumatism of unspecified site |
| 310780015 | Palindromic rheumatism of the upper arm |
| 310792015 | Arthralgia of unspecified site |
| 310815012 | Arthralgia of other specified site |
| 310847019 | Arthralgia of other tarsal joint |
| 311036019 | Arthropathies NOS |
| 311197011 | Cervical spinal stenosis secondary to other disease |
| 311229014 | Spinal stenosis of unspecified region |
| 311236010 | Thoracic spinal stenosis secondary to other disease |
| 311267012 | Sacral ankylosis NOS |
| 311496011 | Fibrosing alveolitis associated with rheumatoid arthritis |
| 311767011 | Other specified nonarticular rheumatism |
| 312518013 | [X]Other seropositive rheumatoid arthritis |
| 312520011 | [X]Other specified rheumatoid arthritis |
| 312534011 | [X]Seropositive rheumatoid arthritis, unspecified |
| 312804012 | Spinal meningocele NOS |
| 312946016 | Other specified spinal cord anomalies |
| 320142012 | Other forearm sprain |
| 320144013 | Forearm sprain NOS |
| 320169013 | Wrist sprain NOS |
| 320181012 | Hand sprain NOS |
| 320235016 | Other thigh sprain |
| 320236015 | Hip sprain NOS |
| 320257011 | Leg sprain NOS |
| 320259014 | Ankle sprain, unspecified |
| 320548016 | Jaw sprain NOS |
| 320562011 | Sternum sprain unspecified |
| 320568010 | Sprain of pelvis, unspecified |
| 320579017 | Other and ill-defined sprains and strains NOS |
| 323940016 | Other face and neck injuries NOS |
| 325375012 | [X]Other multiple injuries of abdomen, lower back and pelvis |
| 359168010 | Ankle arthritis NOS |
| 378021011 | Congenital deformity of musculoskeletal system NEC |
| 400187015 | Osteoarthritis NOS, of the lower leg |
| 400192018 | Unspecified polyarthropathy or polyarthritis NOS |
| 400248014 | Arthralgia of the forearm |
| 400941013 | Congenital musculoskeletal anomalies NOS |
| 402918012 | Neck sprain, unspecified |
| 402920010 | Sprains and strains NOS |
| 402974016 | Late effect of sprain without mention of tendon injury |
| 450558019 | Polyneuropathy unspecified |
| 1209790019 | Arthritis of spine |
| 2474635010 | Rheumatology management plan given |
| 2476044012 | Osteoarthritis of spine NOS |
| 28251000006113 | Other infect/parasit dis with arthropathy of the forearm |
| 28261000006110 | Other infect/parasit dis with arthropathy of the hand |
| 28271000006115 | Other infect/parasit dis with arthropathy of the lower leg |
| 28281000006117 | Other infect/parasit dis with arthropathy of the upper arm |
| 29251000006116 | Other infect/parasit dis with arthropathy of ankle and foot |
| 31041000006113 | Osteoarthritis NOS, of distal radio-ulnar joint |
| 92851000006118 | Traumatic arthropathy of tibio-fibular joint |
| 130611000006116 | Sprain finger |
| 130901000006112 | Sprain radio-lunate ligament |
| 130941000006114 | Sprain scapho-trapezium ligament |
| 130951000006111 | Sprain short intrinsic ligament non-specific |
| 131051000006117 | Sprain thumb, interphalangeal joint, non specific |
| 131111000006119 | Sprain ulno-lunate ligament |
| 131231000006119 | Sprain, coraco-clavicular ligament |
| 135161000006118 | Spinal meningeal adhesions |
| 168291000006116 | Rheumat.dis.treatment changed |
| 168661000006110 | Rheumatism - gonococcal |
| 168751000006115 | Rheumatoid arthritis and other inflammatory polyarthropathy |
| 217691000006119 | Postdysenteric reactive arthropathy of the ankle and foot |
| 219611000000112 | Osteoarthritis cervical spine |
| 221481000000114 | Crystal arthritis |
| 222451000000113 | Headache - post traumatic |
| 264481000006111 | Oligoarticular osteoarthritis, unspecified, of shoulder |
| 264501000006118 | Oligoarticular osteoarthritis, unspecified, other spec sites |
| 265861000006116 | Oligoarticular osteoarthritis, unspecified, multiple sites |
| 363841000006110 | [X]Arthropathy in hypersensitivity reactions CE |
| 363861000006114 | [X]Arthropathy in other blood disorders CE |
| 377471000006118 | [X]Disl'n sprain/strain unsp joint&ligamt upr limb lvl unsp |
| 377511000006111 | [X]Dislocat/sprains/strains involv oth comb of body regions |
| 405421000006113 | [X]Oth specified acquired deformities/musculoskeletal system |
| 412771000006110 | [X]Other postinfectious arthropathies in diseases CE |
| 422931000006116 | [X]Polyneuropathy in infectious+parasitic diseases CE |
| 422981000006115 | [X]Polyneuropathy/other musculoskeletal disorders CE |
| 424491000006118 | [X]Reactive arthropathy in other diseases CE |
| 427441000006117 | [X]Sprain & strain of oth & unspecif parts of wrist & hand |
| 427491000006114 | [X]Sprain/strain of joint/ligam of oth & unsp part of neck |
| 483991000006118 | Ankylosis of other CMC joint |
| 484091000006115 | Ankylosis of the hip joint |
| 484111000006112 | Ankylosis of the knee joint |
| 484121000006116 | Ankylosis of the shoulder joint |
| 492061000006117 | Arthralgia of tibio-fibular joint |
| 492101000006119 | Arthritis associated with other disease, 1st MTP joint |
| 492191000006114 | Arthritis associated with other disease, lesser MTP joint |
| 492251000006111 | Arthritis associated with other disease, sternoclavic joint |
| 492281000006115 | Arthritis associated with other disease, tibio-fibular joint |
| 492781000006112 | Arthropathy due to haemophilia |
| 493021000006115 | Arthropathy NOS |
| 493161000006113 | Arthropathy with other bacterial disease, of hand |
| 493201000006119 | Arthropathy with other bacterial disease, of pelvic/thigh |
| 493211000006116 | Arthropathy with other bacterial disease, of shoulder region |
| 493221000006112 | Arthropathy with other bacterial disease, of unspec site |
| 493231000006110 | Arthropathy with other bacterial disease, of upper arm |
| 538811000006114 | Carpal instability, dorsal subluxation |
| 544811000006116 | Cervico-thoracic ankylosis |
| 556181000006113 | Chronic post-rheumatic arthropathy |
| 567791000006119 | Closed spinal subluxation with cervical cord lesion, unspec |
| 567861000006117 | Closed spinal subluxation with thoracic cord lesion, unspec |
| 583951000006116 | Congenital musculoskeletal deformities |
| 598231000006114 | Costal cartilage sprain |
| 601221000006114 | Crush inj of oth & unspecif parts of abdom/low back/pelv |
| 666351000006116 | Familial neuropathic amyloid |
| 693491000006111 | Musculoskeletal or connective tissue diseases OS |
| 694101000006113 | Muscle injury / strain |
| 750731000006111 | Late effect of musculoskeletal/connective tissue injury NOS |
| 755441000006119 | Juvenile rheumatoid arthritis - Still's disease |
| 779361000006114 | Infective arthritis NOS, of distal radio-ulnar joint |
| 779391000006118 | Infective arthritis NOS, of IP joint of toe |
| 779471000006110 | Infective arthritis NOS, of sacro-iliac joint |
| 780201000006117 | Infective arthritis NOS, of 1st MTP joint |
| 828201000006113 | Horton's (histamine) neuralgia |
| 309700017 | Crystal arthropathy NOS, of the shoulder region |
| 309708012 | Crystal arthropathy NOS, of sternoclavicular joint |
| 309731012 | Crystal arthropathy NOS, of multiple sites |
| 603481000006111 | Crystal arthropathy NOS, of DIP joint of finger |
| 603491000006114 | Crystal arthropathy NOS, of distal radio-ulnar joint |
| 603541000006116 | Crystal arthropathy NOS, of lesser MTP joint |
| 603551000006119 | Crystal arthropathy NOS, of MCP joint |
| 778621000006116 | Inflammatory polyneuropathy, unspecified |
| 913981000006115 | Insulin dependent diab mell with neuropathic arthropathy |
| 310055010 | Traumatic arthropathy of the lower leg |
| 400225016 | Joint ankylosis of the lower leg |
| 989021000006118 | Other peripheral neuropathy |
| 130991000006117 | Sprain thumb |
| 212321000006111 | Postprocedural musculoskeletal disorder, unspecified |
| 461576019 | [Q] Central spinal stenosis |
| 461007015 | [V]Unspecified psychological or physical strain |
| 363831000006117 | [X]Arthropathies/oth endocrin,nutritionl+metabolic disorders |
| 416711000006117 | [X]Other superfic injuries of abdomen, lower back & pelvis |
| 297571012 | Other toxic agent polyneuropathy |
| 309522010 | Arthropathy associated with other viral diseases |
| 309527016 | Arthropathy with other viral disease, of hand |
| 309533013 | Arthropathy associated with other viral disease NOS |
| 309915010 | Localised, secondary osteoarthritis of unspecified site |
| 309930018 | Localised, secondary osteoarthritis of the lower leg |
| 309949019 | Localised osteoarthritis, unspecified, of the upper arm |
| 309971012 | Localised osteoarthritis, unspecified, NOS |
| 310109010 | Transient arthropathy of the forearm |
| 310550011 | Ankylosis of other pelvic joint |
| 312494011 | Other specified musculoskeletal disorders |
| 130571000006114 | Sprain & strain of other and unspecified parts of thorax |
| 217751000006115 | Postdysenteric reactive arthropathy of the upper arm |
| 333341000006111 | [SO]Specified part of musculoskeletal system NEC |
| 493241000006117 | Arthropathy with other viral disease, of ankle and foot |
| 493281000006111 | Arthropathy with other viral disease, of multiple sites |
| 493291000006114 | Arthropathy with other viral disease, of other spec site |
| 493301000006110 | Arthropathy with other viral disease, of pelvic region/thigh |
| 493311000006113 | Arthropathy with other viral disease, of shoulder region |
| 493321000006117 | Arthropathy with other viral disease, of unspecified site |
| 312516012 | [X]Inflammatory polyarthropathies |
| 889871000006117 | Osteoarthritis -shoulder joint |
| 889921000006110 | Osteoarthritis - knee joint |
| 889941000006115 | Osteoarthritis - other joint |
| 890411000006115 | Ankylosis - wrist joint |
| 890461000006117 | Ankylosis - other joint |
| 891021000006111 | Back disorder/symptom NOS |
| 891571000006115 | Rheumatism/fibrositis NOS |
| 891601000006110 | Rheumatism NOS - hip |
| 891641000006112 | Rheumatism NOS - shoulder |
| 891681000006118 | Myalgia/myositis - lower leg |
| 891691000006115 | Myalgia/myositis -pelvis/thigh |
| 891701000006115 | Myalgia/myositis - hand |
| 891711000006117 | Myalgia/myositis - fore-arm |
| 891721000006113 | Myalgia/myositis - upper arm |
| 891841000006113 | Neuralgia/neuritis - multiple |
| 891851000006110 | Neuralgia/neuritis NOS |
| 892711000006110 | Musculoskeletal problems NOS |
| 892731000006116 | Musculoskeletal disease NOS |
| 894711000006115 | Headache symptom NOS [D] |
| 896181000006117 | Sprain - upper arm |
| 989341000006110 | Osteoarthritis - elbow joint |
| 989351000006112 | Osteoarthritis - wrist joint |
| 990171000006111 | Sprain - lower leg |
| 990331000006110 | Sprain - upper arm |
| 991371000006119 | Other sprains NOS |
| 991401000006116 | Sprained knee NOS |
| 1464018 | Monoarthritis |
| 297165012 | Disease related peripheral neuropathy |
| 297338014 | Cluster headache syndrome |
| 297427010 | Trigeminal neuralgia |
| 297528015 | Idiopathic peripheral neuropathy |
| 299360019 | Mononeuropathy of lower limb |
| 309400018 | Arthropathy |
| 309972017 | Osteoarthritis of multiple joints |
| 310012011 | Osteoarthritis of sternoclavicular joint |
| 310013018 | Osteoarthritis of acromioclavicular joint |
| 310028018 | Osteoarthritis of talonavicular joint |
| 310855014 | Arthralgia |
| 311041010 | Sacroiliitis |
| 311045018 | Spondylitis |
| 311688013 | Rheumatism |
| 311707014 | Neuritis |
| 312504018 | Musculoskeletal disorder |
| 312510018 | Streptococcal arthritis |
| 312521010 | Psoriasis with arthropathy |
| 312703010 | Hypertrophic osteoarthropathy |
| 312729010 | Musculoskeletal and connective tissue disorder |
| 317264010 | [D]Headache |
| 320143019 | Elbow sprain |
| 320147018 | Wrist sprain |
| 320220017 | Sprain of wrist and/or hand |
| 320234017 | Sprain of hip |
| 320318018 | Back sprain |
| 320560015 | Sprained rib |
| 325451011 | Sprain of knee |
| 359167017 | Arthritis of foot |
| 359388019 | Osteoarthrosis of the carpometacarpal joint of the thumb |
| 391249010 | Ligament sprain |
| 400188013 | Localised, primary osteoarthritis of the ankle and/or foot |
| 400263012 | Arthralgia of the ankle and/or foot |
| 400314010 | Myalgia |
| 400878010 | Congenital anomaly of musculoskeletal system |
| 406699016 | Ankle instability |
| 18051000006110 | Osteoarthritis of proximal interphalangeal joint |
| 41301000006112 | Osteoarthritis of first metatarsophalangeal joint |
| 87721000006119 | Trigeminal nerve disorder |
| 92671000006112 | Traumatic arthropathy of lesser metatarsophalangeal joint |
| 106921000006111 | Thoracic back sprain |
| 130631000006110 | Sprain finger, distal interphalangeal joint, ulnar collateral ligament |
| 130641000006117 | Sprain finger, metacarpophalangeal joint, radial collateral ligament |
| 130651000006115 | Sprain finger, metacarpophalangeal joint, ulnar collateral ligament |
| 130671000006113 | Sprain finger, proximal interphalangeal joint, ulnar collateral ligament |
| 130711000006112 | Sprain finger, proximal interphalangeal joint, nonspecific |
| 130721000006116 | Strain of gastrocnemius tendon |
| 130931000006116 | Sprain of scapholunate ligament |
| 131171000006111 | Sprain of wrist |
| 131391000006114 | Sprain of interphalangeal joint of toe |
| 131401000006111 | Sprain of lateral collateral ligament of knee |
| 131461000006112 | Strain of patellar tendon |
| 131481000006119 | Sprain, posterior sacroiliac ligament |
| 131551000006111 | Strain of subscapularis tendon |
| 131561000006113 | Strain of supraspinatus tendon |
| 131591000006117 | Strain of Achilles tendon |
| 140551000006117 | Arthritis of shoulder region joint |
| 155721000006112 | Injury of sciatic nerve |
| 159761000006115 | Sprain of sacrococcygeal ligament |
| 159821000006119 | Sprain of ligament of sacroiliac joint |
| 162101000006115 | Rheumatoid arthritis of lesser metatarsophalangeal joint |
| 162111000006117 | Rheumatoid arthritis of metacarpophalangeal joint |
| 162641000006113 | Sprain of costal cartilage |
| 168241000006113 | Rheumatology follow-up assessment |
| 168261000006112 | Rheumatology symptom change |
| 192631000006118 | Reactive arthritis |
| 192711000006116 | Reactive arthropathy of interphalangeal joint of toe |
| 192731000006110 | Reactive arthropathy of lesser metatarsophalangeal joint |
| 198881000006116 | Psoriatic spondylitis |
| 211231000006113 | Post-herpetic polyneuropathy |
| 219261000000112 | Crystal arthropathy |
| 221511000000115 | Osteoarthritis of hip |
| 359201000006116 | Tension-type headache |
| 394571000006116 | Chronic arthritis of juvenile onset |
| 428461000006117 | [X]Symptoms and signs involving the nervous and musculoskeletal systems |
| 483971000006119 | Ankylosis of metatarsophalangeal joint |
| 484031000006119 | Ankylosis of proximal interphalangeal joint |
| 491791000006117 | Arthralgia of 1st metatarsophalangeal joint |
| 491861000006115 | Arthralgia of interphalangeal joint of toe |
| 491891000006111 | Arthralgia of metacarpophalangeal joint |
| 492521000006116 | Arthropathy associated with non-infective gastrointestinal disorders |
| 492911000006119 | Arthropathy in Behcet's syndrome of the pelvic region and thigh |
| 567631000006110 | Closed spinal dislocation with posterior cervical cord lesion |
| 623361000006116 | Dislocations, sprains and strains involving thorax with lower back and pelvis |
| 623391000006112 | Dislocations, sprains and strains involving multiple regions of lower limb(s) |
| 623401000006114 | Dislocations, sprains and strains involving multiple regions of upper limb(s) and lower limb(s) |
| 777831000006118 | Injury of nerves and lumbar spinal cord at abdomen, lower back and pelvis level |
| 800731000006117 | Generalised osteoarthritis |
| 800751000006112 | Polyarticular osteoarthritis |
| 819781000006110 | Arthropathy associated with helminthiasis |
| 1715221000006117 | Cervicogenic headache |
| 1716421000006114 | Chronic headache disorder |
| 1747081000000119 | History of migraine with aura |
| 1786091000006118 | Benign exertion headache |
| 1871131000006116 | Lower back injury |
| 309703015 | Crystal arthropathy of hand |
| 309706011 | Crystal arthropathy of ankle AND/OR foot |
| 309707019 | Crystal arthropathy of shoulder region |
| 309718019 | Crystal arthropathy of hip |
| 309722012 | Crystal arthropathy of ankle AND/OR foot |
| 318040019 | [X]Other chronic pain |
| 16911000006114 | Rheumatoid arthritis with organ / system involvement |
| 221661000006114 | Inflammatory polyarthritis |
| 451461014 | Seropositive erosive rheumatoid arthritis |
| 2839291014 | Disease activity score 28 joint in rheumatoid arthritis |
| 310944013 | Arthropathy of ankle and/or foot |
| 1222484010 | Musculoskeletal symptom |
| 422921000006119 | [X]Polyneuropathies and other disorders of the peripheral nervous system |
| 890401000006118 | Ankylosis - elbow joint |
| 890441000006116 | Ankylosis - knee joint |
| 891581000006117 | Rheumatism NOS - ankle/foot |
| 891591000006119 | Rheumatism NOS - knee |
| 891631000006119 | Rheumatism NOS - elbow |
| 891821000006118 | Neuralgia/neuritis - upper arm |
| 896251000006117 | Sprain - hand NOS |
| 896451000006116 | Sprain - back |
| 896511000006116 | Other sprains NOS |
| 990321000006112 | Sprained shoulder |
| 309841017 | Other specified inflammatory polyarthropathy |
| 309919016 | Localised, secondary osteoarthritis of the upper arm |
| 309945013 | Localised osteoarthritis, unspecified, of unspecified site |
| 427451000006115 | [X]Sprain & strain of other and unspecified parts of thorax |
| 736701000006117 | Localised osteoarthritis, unspecified, of other spec site |
| 461578018 | [Q] Central and lateral spinal stenosis |
| 377571000006119 | [X]Dislocation, sprain and strain of unspecif body region |
| 309710014 | Crystal arthropathy NOS, of acromioclavicular joint |
| 772131000006111 | Insulin dependent diab mell with neuropathic arthropathy |
| 883171000006110 | Other peripheral neuropathy |
| 359071000006116 | [X] Polyneuropathy, unspecified |
| 366601000006119 | [X]Autonomic neuropathy/endocrine+metabolic diseases CE |
| 408441000006111 | [X]Other congenital malforms of the musculoskeletal system |
| 422971000006118 | [X]Polyneuropathy/other endocrine+metabolic diseases CE |
| 423971000006114 | [X]Psychogenic headache |
| 484071000006116 | Ankylosis of the ankle joint |
| 492221000006119 | Arthritis associated with other disease, PIP joint of finger |
| 493181000006115 | Arthropathy with other bacterial disease, of multiple sites |
| 570661000006112 | Cls spinal # with incomplete thoracid cord lesion, T7-12 NOS |
| 603521000006111 | Crystal arthropathy NOS, of IP joint of toe |
| 603721000006119 | Crystal arthropathy NOS, of tibio-fibular joint |
| 623781000006113 | Dislocations, sprains & strains involv multiple body regions |
| 631181000006113 | Drug-induced headache, not elsewhere classified |
| 673591000006119 | Nodular fibrositis of chronic rheumatic disease |
| 89511000006113 | Tuberculosis of meninges and central nervous system |
| 130911000006110 | Sprain radio-scapho-capitate ligament |
| 131211000006113 | Sprain, anterior sacro-iliac ligament |
| 131451000006110 | Sprain, mid tarsal joint |
| 211281000006114 | Postimmunization arthropathy |
| 211341000006110 | Postinfective arthropathy in syphilis |
| 214871000006114 | Polyneuropathy due to drugs |
| 217711000006116 | Postdysenteric reactive arthropathy of the hand |
| 299346016 | [X]Vascular headache, not elsewhere classified |
| 309463019 | Pyogenic arthritis of other specified sites |
| 309484012 | Arthropathy in Behcet's syndrome of the upper arm |
| 309521015 | Arthropathy associated with other bacterial disease NOS |
| 309534019 | Arthropathy associated with mycoses, of unspecified site |
| 309536017 | Arthropathy associated with mycoses, of the upper arm |
| 309594016 | Infective arthritis NOS |
| 309595015 | Infective arthritis NOS, of unspecified site |
| 309598018 | Infective arthritis NOS, of the forearm |
| 309599014 | Infective arthritis NOS, of the hand |
| 309620013 | Infective arthritis NOS, of talonavicular joint |
| 309621012 | Infective arthritis NOS, of other tarsal joint |
| 309693017 | Other crystal arthropathies of the ankle and foot |
| 309828016 | Juvenile rheumatoid arthritis NOS |
| 309899016 | Localised, primary osteoarthritis of other specified site |
| 309976019 | Oligoarticular osteoarthritis, unspecified, of forearm |
| 310051018 | Traumatic arthropathy of the upper arm |
| 310091015 | Allergic arthritis NOS |
| 310150019 | Unspecified polyarthropathy of the upper arm |
| 310151015 | Unspecified polyarthropathy of the forearm |
| 310166017 | Unspecified monoarthritis of unspecified site |
| 310171012 | Unspecified monoarthritis of the pelvic region and thigh |
| 310173010 | Unspecified monoarthritis of the ankle and foot |
| 310190016 | Other specified arthropathy of other specified site |
| 310784012 | Palindromic rheumatism of the lower leg |
| 312513016 | [X]Other reactive arthropathies |
| 312959012 | Other specified spinal cord anomalies NOS |
| 314955017 | Other congenital musculoskeletal anomalies |
| 320281016 | Ankle and foot sprain NOS |
| 320550012 | Thyroid region sprain, unspecified |
| 378099017 | Endemic polyarthritis |
| 400157014 | Pyogenic arthritis of the upper arm |
| 400184010 | Osteoarthritis NOS, of the forearm |
| 400194017 | Arthropathy NOS |
| 400195016 | Arthropathy NOS, of the upper arm |
| 400196015 | Arthropathy NOS, of the forearm |
| 402909010 | Other shoulder sprain |
| 158050019 | Polymyalgia |
| 252315012 | Back pain without radiation NOS |
| 295346011 | Psychogenic musculoskeletal symptoms NOS |
| 297519016 | Hereditary peripheral neuropathy NOS |
| 55627011 | Psoriatic arthropathy |
| 311696015 | Myalgia and myositis unspecified |
| 1174311000000111 | Arthropathy in cystic fibrosis |
| 895321000006111 | #Cervical spine + cord lesion |
| 895341000006116 | #Lumbar spine + cord lesion |
| 896221000006114 | Sprain - hand |
| 162261000006114 | Rheumatoid factor |
| 359289013 | Arthropathy following intestinal bypass |
| 359312010 | Juvenile ankylosing spondylitis |
| 391100012 | Leg sprain |
| 391101011 | Back sprain excluding lumbosacral |
| 400159012 | Pyogenic arthritis of the pelvic region and thigh |
| 400250018 | Arthralgia of the lower leg |
| 426510015 | Rheumatoid arthritis - multiple joint |
| 309460016 | Pneumococcal arthritis and polyarthritis |
| 309535018 | Arthropathy associated with mycoses, of the shoulder region |
| 309562014 | Reactive arthropathy of sternoclavicular joint |
| 309680010 | Gouty arthritis of the ankle and foot |
| 309802019 | Rheumatoid arthritis of ankle |
| 309827014 | Monarticular juvenile rheumatoid arthritis |
| 309923012 | Localised, secondary osteoarthritis of the hand |
| 310073013 | Traumatic arthropathy of subtalar joint |
| 310090019 | Allergic arthritis of multiple sites |
| 310096013 | Climacteric arthritis of the shoulder region |
| 310782011 | Palindromic rheumatism of the hand |
| 311196019 | Iatrogenic cervical spinal stenosis |
| 311237018 | Idiopathic lumbar spinal stenosis |
| 311290019 | Cervical spine instability |
| 311694017 | Hand rheumatism |
| 311695016 | Rheumatism or fibrositis NOS |
| 312801016 | Cervical spinal meningocele |
| 319716011 | Closed spinal dislocation with central cervical cord lesion |
| 319768016 | Closed spinal dislocation with complete lumbar cord lesion |
| 319769012 | Closed spinal dislocation with anterior lumbar cord lesion |
| 320184016 | Sprain thumb, carpometacarpal joint |
| 320210010 | Sprain tendon wrist or hand |
| 320228012 | Ischiocapsular sprain |
| 320250013 | Sprain, plantaris tendon |
| 320279018 | Sprain, extensor tendon, foot |
| 320551011 | Cricoarytenoid sprain |
| 320552016 | Cricothyroid sprain |
| 293128014 | Tuberculous arthritis |
| 297540015 | Polyneuropathy in collagen vascular disease |
| 297558014 | Polyneuropathy in hypoglycaemia |
| 303172010 | Irritable bowel syndrome with diarrhoea |
| 252313017 | Back pain worse on sneezing |
| 253119015 | Throbbing headache |
| 253120014 | Shooting headache |
| 253121013 | Morning headache |
| 9300010 | Sinus headache |
| 16067019 | Familial amyloid polyneuropathy, Iowa type |
| 16455010 | Gouty neuritis |
| 29902012 | Median nerve neuritis |
| 93295014 | Common migraine |
| 93296010 | Atypical migraine |
| 96705013 | Traumatic arthropathy |
| 104572011 | Arthropathy in Behcet's syndrome |
| 111967016 | Instability of joint |
| 149683011 | Tuberculosis of cerebral meninges |
| 158459012 | Chronic paroxysmal hemicrania |
| 166489012 | Unilateral headache |
| 477671011 | Sarcoid arthropathy |
| 485678014 | Arthritis in Lyme disease |
| 498448012 | Neuropathic foot ulcer |
| 499415017 | Behcet's syndrome arthropathy |
| 507743013 | Hand sprain |
| 1222298017 | Arthritis due to rubella |
| 1230712018 | Foot sprain |
| 1235653017 | British type amyloid polyneuropathy |
| 1488516017 | Rheumatology |
| 1494989015 | Lumbosacral sprain |
| 1776248011 | Osteoarthritis |
| 1786700015 | Periarthritis of shoulder |
| 2472447010 | Juvenile rheumatoid arthritis |
| 309695012 | Crystal arthropathy of multiple sites |
| 309829012 | Juvenile arthritis |
| 310170013 | Monoarthritis of hand |
| 311398011 | Periarthritis |
| 317098012 | Chronic intractable pain |
| 320130019 | Sprain of shoulder |
| 325470018 | Sprain of foot |
| 359169019 | Arthritis of knee |
| 359170018 | Arthritis of hip |
| 359399017 | Primary generalised osteoarthrosis |
| 402917019 | Sprain of spinal ligament |
| 40781000006114 | Osteopathies, chondropathies and acquired musculoskeletal deformities |
| 99281000006115 | Toxic neuropathy, NOS |
| 131061000006115 | Sprain thumb, metacarpophalangeal joint nonspecific |
| 131441000006113 | Sprain, metatarsophalangeal joint |
| 162081000006111 | Rheumatoid arthritis of interphalangeal joint of toe |
| 162211000006111 | Rheumatoid arthritis screen |
| 168301000006115 | Rheumatology disorder treatment started |
| 192671000006115 | Reactive arthropathy of distal interphalangeal joint of finger |
| 327591000006117 | Musculoskeletal system |
| 483931000006117 | Ankylosis of distal interphalangeal joint |
| 483961000006114 | Ankylosis of metacarpophalangeal joint |
| 483981000006116 | Ankylosis of joint of multiple sites |
| 484061000006111 | Ankylosis of the first carpometacarpal joint |
| 484141000006111 | Ankylosis of the superior radioulnar joint |
| 491881000006113 | Arthralgia of lesser metatarsophalangeal joint |
| 491941000006112 | Arthralgia of sacroiliac joint |
| 492581000006117 | Arthropathy related to infection |
| 492631000006119 | Arthropathy associated with mycoses, of the pelvic region and thigh |
| 492971000006111 | Arthropathy associated with nonspecific urethritis |
| 497071000006114 | Atlantoaxial instability |
| 501991000006110 | Back pain |
| 710161000006114 | Sprain of ligament of finger |
| 253123011 | Finding of headache character |
| 299375010 | Inflammatory polyneuropathy |
| 309616014 | Septic arthritis of knee |
| 309711013 | Crystal arthropathy of elbow |
| 309713011 | Crystal arthropathy of wrist |
| 309748016 | Arthropathy associated with a haematological disorder |
| 101001000006111 | Sprain of thumb |
| 320131015 | Sprain of upper extremity |
| 362631000006114 | [X]Additional musculoskeletal and connective tissue disease classification terms |
| 297404019 | Meninges disorder NEC |
| 310554019 | Ankylosis of other tarsal joint |
| 890041000006110 | Traumatic arthritis NOS |
| 891611000006113 | Rheumatism NOS - hand |
| 891671000006116 | Myalgia/myositis - ankle/foot |
| 891751000006116 | Myalgia/myositis NOS |
| 891781000006112 | Neuralgia/neuritis - lower leg |
| 892721000006119 | Other musculoskelet/connectiv |
| 895361000006117 | #Spine NOS + cord lesion |
| 896191000006119 | Sprained shoulder |
| 896461000006119 | Other sprains |
| 933201000006114 | Benign coital headache |
| 990341000006117 | Sprained elbow |
| 990361000006118 | Sprain - wrist |
| 312509011 | [X]Infectious arthropathies |
| 391113018 | Back dislocation NOS |
| 402910017 | Other upper arm sprain |
| 299811000006115 | [D]Musculosc xray/scan abn NOS |
| 377501000006113 | [X]Dislocat/sprain/strain unsp joint/ligam leg, level unsp |
| 779411000006118 | Infective arthritis NOS, of lesser MTP joint |
| 779461000006115 | Infective arthritis NOS, of PIP joint of finger |
| 736781000006114 | Localised osteoarthritis, unspecified, pelvic region/thigh |
| 309812014 | Rheumatoid bursitis |
| 491831000006112 | Arthralgia of distal radio-ulnar joint |
| 492131000006110 | Arthritis associated with other disease, DIP joint of finger |
| 492231000006116 | Arthritis associated with other disease, sacro-iliac joint |
| 538801000006111 | Carpal instability, D.I.S.I. |
| 405142017 | Unspecified polyarthropathy or polyarthritis |
| 2472449013 | Juvenile seronegative polyarthritis |
| 18121000006117 | Osteoarthritis NOS, of tibio-fibular joint |
| 29271000006114 | Other infect/parasit dis with arthropathy of other spec site |
| 31091000006116 | Osteoarthritis NOS, of lesser MTP joint |
| 106831000006116 | Thoracic spinal hydromeningocele |
| 130551000006116 | Sprain & strain of oth & unsp parts of lumb spine & pelv |
| 130601000006119 | Sprain dorsal radio-carpal ligament |
| 130701000006114 | Sprain finger, metacarpophalangeal joint, non specific |
| 130921000006119 | Sprain radio-scapho-lunate ligament |
| 131131000006113 | Sprain volar radio-carpal ligament non-specific |
| 131411000006114 | Sprain, knee joint, medial collateral |
| 168271000006117 | Rheumat. treatment change |
| 192771000006113 | Reactive arthropathy of sacro-iliac joint |
| 192821000006117 | Reactive arthropathy of tibio-fibular joint |
| 215551000000114 | Sero negative polyarthritis |
| 265871000006111 | Oligoarticular osteoarthritis, unspecified, of ankle/foot |
| 297162010 | Idiopathic peripheral autonomic neuropathy NOS |
| 297336013 | Common migraine NOS |
| 297352011 | Other forms of migraine |
| 309491010 | Arthropathy in Behcet's syndrome of other specified sites |
| 309544017 | Arthropathy associated with mycoses NOS |
| 309577018 | Reactive arthropathy of other tarsal joint |
| 309596019 | Infective arthritis NOS, of the shoulder region |
| 309602016 | Infective arthritis NOS, of the ankle and foot |
| 309619019 | Infective arthritis NOS, of subtalar joint |
| 309683012 | Gouty arthritis NOS |
| 309696013 | Other crystal arthropathies of other specified sites |
| 309698014 | Crystal arthropathy NOS |
| 309702013 | Crystal arthropathy NOS, of the forearm |
| 309705010 | Crystal arthropathy NOS, of the lower leg |
| 309732017 | Crystal arthropathy NOS, of other specified site |
| 309773016 | Arthritis associated with other disease, knee |
| 309775011 | Arthritis associated with other disease, ankle |
| 309777015 | Arthritis associated with other disease, talonavicular joint |
| 309824019 | Juvenile rheumatoid arthropathy unspecified |
| 309884016 | Localised, primary osteoarthritis of unspecified site |
| 309888018 | Localised, primary osteoarthritis of the upper arm |
| 309889014 | Localised, primary osteoarthritis of the forearm |
| 309941016 | Localised, secondary osteoarthritis NOS |
| 309965018 | Localised osteoarthritis, unspecified, of the ankle and foot |
| 309983014 | Osteoarthritis of more than one site, unspecified, NOS |
| 309987010 | Osteoarthritis NOS, of unspecified site |
| 310024016 | Osteoarthritis NOS, of knee |
| 310079012 | Traumatic arthropathy NOS |
| 310101013 | Climacteric arthritis of the lower leg |
| 310103011 | Climacteric arthritis of other specified site |
| 310149019 | Unspecified polyarthropathy of the shoulder region |
| 310191017 | Other specified arthropathy of multiple sites |
| 310216015 | Arthropathy NOS, of other specified site |
| 310518018 | Joint ankylosis of unspecified site |
| 310788010 | Palindromic rheumatism NOS |
| 311261013 | Thoracic and lumbosacral neuritis NOS |
| 312960019 | Spinal cord anomalies NOS |
| 320305014 | Neck sprain NOS |
| 320557010 | Rib sprain unspecified |
| 345456011 | Neuritis ulnar nerve |
| 391057017 | Muscle sprain NOS |
| 400182014 | Osteoarthritis NOS |
| 400183016 | Osteoarthritis NOS, of the upper arm |
| 496410014 | ME - Myalgic encephalomyelitis |
| 496413011 | Myalgic encephalomyelitis |
| 1757331000000118 | Trigeminal autonomic cephalalgia |
| 875231000006113 | Therapeutic asp.-musculoskelet |
| 896351000006111 | Sprain - Achilles tendon |
| 896501000006119 | Sprained symphisis pubis |
| 311711000000114 | Medication overuse headache |
| 309483018 | Arthropathy in Behcet's syndrome of the shoulder region |
| 309490011 | Arthropathy in Behcet's syndrome of multiple sites |
| 309566012 | Reactive arthropathy of wrist |
| 309681014 | Gouty arthritis of multiple sites |
| 309836013 | Juvenile arthritis in ulcerative colitis |
| 309885015 | Localised, primary osteoarthritis of the shoulder region |
| 310060014 | Traumatic arthropathy of sternoclavicular joint |
| 310061013 | Traumatic arthropathy of acromioclavicular joint |
| 310100014 | Climacteric arthritis of the pelvic region and thigh |
| 310519014 | Joint ankylosis of the shoulder region |
| 310540010 | Elbow joint ankylosis |
| 310556017 | Ankylosis of toe joint |
| 310783018 | Palindromic rheumatism of the pelvic region and thigh |
| 310785013 | Palindromic rheumatism of the ankle and foot |
| 310822016 | Arthralgia of elbow |
| 310839019 | Arthralgia of ankle |
| 310842013 | Arthralgia of subtalar joint |
| 312011010 | Hypertrophic pulmonary osteoarthropathy |
| 312350016 | Chronic instability of knee |
| 312790015 | Cervical spinal hydromeningocele |
| 319750019 | Open spinal dislocation with anterior cervical cord lesion |
| 319751015 | Open spinal dislocation with central cervical cord lesion |
| 319867016 | Closed spinal subluxation with central thoracic cord lesion |
| 320110018 | Sprains and strains of joints and adjacent muscles |
| 320118013 | Sprain, shoulder joint, anterior |
| 320120011 | Sprain, biceps tendon |
| 320168017 | Sprain dorsal intercarpal ligament |
| 320211014 | Sprain wrist extensors |
| 320213012 | Sprain tendon of thumb |
| 320249013 | Sprain of superior tibiofibular ligament |
| 320263019 | Distal tibiofibular sprain |
| 320303019 | Atlanto-occipital joint sprain |
| 320307018 | Lumbar sprain |
| 320308011 | Lumbar back sprain |
| 324031014 | Multiple open wounds of abdomen, lower back and pelvis |
| 345361012 | Chronic tension-type headache |
| 297164011 | Autonomic neuropathy due to amyloid |
| 297354012 | Complicated migraine |
| 297470010 | Mononeuritis of upper limb and mononeuritis multiplex |
| 297641010 | Myopathy due to rheumatoid arthritis |
| 253103010 | Headache site |
| 28671011 | Allergic arthritis |
| 50336019 | Morton's neuralgia |
| 55628018 | Psoriatic arthritis |
| 56659013 | Lyme arthritis |
| 59922014 | Chronic arthritis |
| 63055014 | Migraine |
| 70659018 | Polyneuropathy |
| 80383012 | Septic arthritis |
| 94456015 | Polyneuritis cranialis |
| 116082011 | Rheumatoid arthritis |
| 500766011 | Neuropathic arthropathy |
| 504520011 | Haemophilic arthropathy |
| 1219615011 | Arthropathy associated with mycoses |
| 1228677017 | Pyogenic arthritis of the shoulder region |
| 1229173011 | Hip pyogenic arthritis |
| 1779323014 | Rheumatoid vasculitis |
| 359268016 | Elbow pyogenic arthritis |
| 359292012 | Seronegative rheumatoid arthritis |
| 359402016 | Osteoarthritis of spinal facet joint |
| 372799017 | Back stiffness |
| 400160019 | Pyogenic arthritis of the lower leg |
| 400161015 | Pyogenic arthritis of the ankle and foot |
| 400251019 | Arthralgia of the ankle and foot |
| 407069017 | C/O - a headache |
| 415888015 | Chronic low back pain |
| 457122015 | Localised, primary osteoarthritis of elbow |
| 459312014 | Type II diabetes mellitus with neuropathic arthropathy |
| 459313016 | Type 2 diabetes mellitus with neuropathic arthropathy |
| 309505013 | Arthropathy associated with bacterial disease |
| 309560018 | Post-infective arthritis |
| 311225015 | Back problem |
| 311260014 | Lumbosacral neuritis |
| 359171019 | Arthritis of wrist |
| 359384017 | Osteoarthritis of foot joint |
| 359385016 | Osteoarthritis of ankle |
| 391075016 | Tendon strain |
| 18061000006112 | Inflammation of sacroiliac joint |
| 31031000006115 | Osteoarthritis of distal interphalangeal joint |
| 60781000006118 | Villonodular synovitis of sacroiliac joint |
| 92641000006116 | Traumatic arthropathy of distal interphalangeal joint of finger |
| 92661000006117 | Traumatic arthropathy of interphalangeal joint of toe |
| 92721000006115 | Traumatic arthropathy of proximal interphalangeal joint of finger |
| 108781000006112 | Ankylosis of temporomandibular joint |
| 131031000006112 | Sprain thumb, metacarpophalangeal joint, ulnar collateral ligament |
| 131181000006114 | Sprain of ligament of acromioclavicular joint |
| 131381000006111 | Strain of infraspinatus tendon |
| 131491000006116 | Strain of quadriceps tendon |
| 131521000006119 | Sprain of shoulder joint |
| 131581000006115 | Sprain, tarsometatarsal joint |
| 168281000006119 | Rheumatology disorder - joints affected |
| 192741000006117 | Reactive arthropathy of metacarpophalangeal joint |
| 217741000000112 | Sinus headache |
| 221521000000114 | Osteoarthritis of knee |
| 235151000006117 | Migrainous neuralgia |
| 491931000006119 | Arthralgia of proximal interphalangeal joint of finger |
| 492541000006111 | Arthropathy associated with another disorder |
| 544821000006112 | Cervicothoracic instability |
| 567841000006116 | Closed spinal subluxation with posterior thoracic cord lesion |
| 616971000006114 | Diabetic Charcot's arthropathy |
| 736911000006116 | Localised, primary osteoarthritis of the pelvic region and thigh |
| 815501000006110 | Arthritis of hand |
| 883141000006119 | Mononeuritis - upper limb |
| 883151000006117 | Other mononeuritis -lower limb |
| 891621000006117 | Rheumatism NOS - wrist |
| 891651000006114 | Rheumatism NOS - multiple |
| 891661000006111 | Rheumatism/fibrositis NOS |
| 891741000006118 | Myalgia/myositis - multiple |
| 891791000006110 | Neuralgia/neurit.-pelvis/thigh |
| 891801000006111 | Neuralgia/neuritis - hand |
| 896391000006117 | Sprain - foot NOS |
| 933131000006112 | Back injury |
| 989361000006114 | Osteoarthritis - hip joint |
| 990101000006117 | Sprained thigh - upper leg |
| 409930017 | Thoracic back sprain |
| 317103014 | [D]Nervous and musculoskeletal symptoms |
| 309727018 | Crystal arthropathy NOS, of other tarsal joint |
| 309805017 | Rheumatoid arthritis of other tarsal joint |
| 312523013 | [X]Other juvenile arthritis |
| 312728019 | [X]Postprocedural musculoskeletal disorder, unspecified |
| 320111019 | Sprain of shoulder and upper arm |
| 492141000006117 | Arthritis associated with other disease, dist rad-uln joint |
| 493001000006113 | Arthropathy in Whipple's disease |
| 493141000006114 | Arthropathy with other bacterial disease, of ankle and foot |
| 538841000006113 | Carpal instability, V.I.S.I. |
| 567621000006112 | Closed spinal dislocation with thoracic cord lesion, unspec |
| 567831000006114 | Closed spinal subluxation with lumbar cord lesion, unspec |
| 570641000006113 | Cls spinal # with incomplete cervical cord lesion, C5-7 NOS |
| 615711000006112 | Detrusor instability |
| 675631000006115 | Neuropathic arthritis |
| 693501000006115 | Musculoskeletal pain - joints |
| 698001000006119 | Mononeuritis lower limb |
| 718001000006112 | Marie - Strumpell spondylitis |
| 779351000006112 | Infective arthritis NOS, of DIP joint of finger |
| 142901000006114 | Sinus headache |
| 162051000006115 | Rheumatoid arthritis of distal radio-ulnar joint |
| 192681000006117 | Reactive arthropathy of distal radio-ulnar joint |
| 376211000006111 | [X]Crystal arthropathy in other metabolic disorders CE |
| 310186018 | Other specified arthropathy of the hand |
| 310192012 | Other specified arthropathy NOS |
| 310781016 | Palindromic rheumatism of the forearm |
| 310786014 | Palindromic rheumatism of other specified site |
| 312789012 | Spinal hydromeningocele, unspecified |
| 314546014 | Congenital musculoskeletal deformity NOS |
| 315764010 | [X]Other specified congenital musculoskeletal deformities |
| 317576014 | [D]Abdominal migraine |
| 320290011 | Other specified sacroiliac sprains |
| 320291010 | Sacroiliac sprain NOS |
| 320554015 | Thyroid region sprain NOS |
| 399418016 | Toxic or inflammatory neuropathy NOS |
| 400315011 | Neuralgia, neuritis or radiculitis NOS |
| 460304013 | [V]Personal history of other musculoskeletal disorders |
| 18191000006115 | Osteoarthritis spine |
| 31071000006117 | Osteoarthritis NOS, of IP joint of toe |
| 41261000006115 | Osteoarthritis and allied disorders |
| 41281000006113 | Osteoarthritis NOS |
| 82841000006116 | Tuberculous arthritis |
| 84391000006111 | Type 1 diabetes mellitus with neuropathic arthropathy |
| 130691000006114 | Sprain finger, distal interphalangeal joint, non specific |
| 130731000006118 | Sprain luno-triquetral ligament |
| 130881000006110 | Sprain proximal radiocarpal ligament non-specific |
| 297429013 | Other trigeminal nerve disorder |
| 297574016 | Other toxic or inflammatory neuropathy |
| 309540014 | Arthropathy associated with mycoses, of the lower leg |
| 309543011 | Arthropathy associated with mycoses, of other specified site |
| 309550010 | Helminthiasis with arthropathy of the upper arm |
| 309603014 | Infective arthritis NOS, of shoulder |
| 309679012 | Gouty arthritis of the lower leg |
| 309776012 | Arthritis associated with other disease, subtalar joint |
| 309850015 | Inflammatory polyarthropathy NOS |
| 309979014 | Oligoarticular osteoarthritis, unspecified, of lower leg |
| 310020013 | Osteoarthritis NOS, of hip |
| 310057019 | Traumatic arthropathy of other specified site |
| 310075018 | Traumatic arthropathy of other tarsal joint |
| 310095012 | Climacteric arthritis of unspecified site |
| 310097016 | Climacteric arthritis of the upper arm |
| 310153017 | Unspecified polyarthropathy of the pelvic region and thigh |
| 310155012 | Unspecified polyarthropathy of the ankle and foot |
| 310168016 | Unspecified monoarthritis of the upper arm |
| 310174016 | Unspecified monoarthritis of other specified site |
| 310175015 | Unspecified monoarthritis NOS |
| 310185019 | Other specified arthropathy of the forearm |
| 158497012 | Ulnar neuritis |
| 990121000006110 | Sprain - lateral knee ligament |
| 990371000006113 | Sprain - hand |
| 990381000006111 | Sprained finger/thumb |
| 889731000006111 | Rheumatoid arthritis NOS |
| 889911000006119 | Osteoarthritis - hip joint |
| 889931000006113 | Osteoarthritis - ankle/foot |
| 896201000006116 | Sprain - fore arm |
| 896211000006118 | Sprained elbow |
| 896271000006110 | Sprained hip |
| 896371000006118 | Sprained foot |
| 896471000006114 | Sprained jaw |
| 2548331017 | Inflammatory polyarthropathy |
| 2674612015 | History of headache |
| 490703010 | Sciatic nerve lesion |
| 493961016 | Familial amyloid polyneuropathy type II |
| 497076011 | Knee sprain |
| 1229552016 | Arthropathy associated with endocrine and metabolic disorder |
| 1234550018 | Congenital spinal cord anomaly |
| 1235278014 | Lumbosacral ankylosis |
| 1488690018 | FH: Ankylosing spondylitis |
| 1777688018 | Muscular headache |
| 2164085015 | Detrusor instability |
| 359266017 | Knee pyogenic arthritis |
| 359273010 | Arthropathy due to parasitic infection |
| 391056014 | Rectus muscle sprain |
| 391098012 | Hamstring sprain |
| 397716013 | FH: Musculoskeletal disease |
| 411549017 | H/O: arthritis |
| 416146018 | Mechanical low back pain |
| 297542011 | Polyneuropathy in disseminated lupus erythematosus |
| 297544012 | Polyneuropathy in rheumatoid arthritis |
| 297557016 | Polyneuropathy in herpes zoster |
| 297563013 | Polyneuropathy in pellagra |
| 309486014 | Arthropathy in Behcet's syndrome of the hand |
| 309538016 | Arthropathy associated with mycoses, of the hand |
| 309541013 | Arthropathy associated with mycoses, of the ankle and foot |
| 309542018 | Arthropathy associated with mycoses, of multiple sites |
| 309561019 | Reactive arthropathy of shoulder |
| 309564010 | Reactive arthropathy of elbow |
| 309677014 | Gouty arthritis of the hand |
| 309678016 | Gouty arthritis of the pelvic region and thigh |
| 309791014 | Rheumatoid arthritis of acromioclavicular joint |
| 309804018 | Rheumatoid arthritis of talonavicular joint |
| 310058012 | Traumatic arthropathy of multiple sites |
| 310074019 | Traumatic arthropathy of talonavicular joint |
| 310435017 | Carpal instability |
| 310539013 | Ankylosis of the elbow joint |
| 310553013 | Ankylosis of the subtalar joint |
| 310779018 | Palindromic rheumatism of the shoulder region |
| 310802015 | Arthralgia of the hand |
| 310819018 | Arthralgia of acromioclavicular joint |
| 310846011 | Arthralgia of talonavicular joint |
| 311293017 | Lumbar spine instability |
| 312788016 | Spinal hydromeningocele |
| 312799019 | Spinal meningocele |
| 312803018 | Lumbar spinal meningocele |
| 312808010 | Lumbar meningomyelocele |
| 319714014 | Closed spinal dislocation with complete cervical cord lesion |
| 319715010 | Closed spinal dislocation with anterior cervical cord lesion |
| 319866013 | Closed spinal subluxation with anterior thoracic cord lesion |
| 320135012 | Sprain, elbow joint, radial collateral ligament |
| 320151016 | Sprain radial collateral ligament |
| 320261017 | Deltoid ligament ankle sprain |
| 320288010 | Sprain, sacrotuberous ligament |
| 320301017 | Cervical anterior longitudinal ligament sprain |
| 320573016 | Complete tear, sacroiliac ligament |
| 250085017 | FH: Rheumatoid arthritis |
| 250086016 | FH: Osteoarthritis |
| 253117018 | Headache character |
| 255925015 | O/E-hands-rheumatoid spindling |
| 7595017 | Migraine with aura |
| 18666015 | Irritable bowel syndrome |
| 30628014 | Congenital deformity of sacroiliac joint |
| 32869011 | Arthritis mutilans |
| 80869018 | Muscle strain |
| 93294013 | Migraine without aura |
| 108062014 | Hereditary peripheral neuropathy |
| 129259017 | Climacteric arthritis |
| 135862013 | Sprain of medial collateral ligament of knee |
| 138588018 | Cervical spinal stenosis |
| 7596016 | Migraine with typical aura |
| 14103017 | Alcohol-induced polyneuropathy |
| 299339013 | Headache disorder |
| 359172014 | Arthritis of elbow |
| 359383011 | Osteoarthritis of toe joint |
| 31101000006110 | Osteoarthritis of metacarpophalangeal joint |
| 106931000006114 | Thoracolumbar ankylosis |
| 131021000006114 | Sprain thumb, metacarpophalangeal joint, radial collateral ligament |
| 159841000006114 | Sacroiliac sprain |
| 164851000006115 | Strain of rotator cuff of shoulder |
| 168761000006118 | Rheumatoid arthritis of 1st metatarsophalangeal joint |
| 192641000006111 | Reactive arthropathy of 1st metatarsophalangeal joint |
| 192761000006118 | Reactive arthropathy of proximal interphalangeal joint of finger |
| 221671000006119 | Polyarthropathy |
| 427481000006111 | Strain of tendon of head and neck |
| 483811000006116 | Pyogenic arthritis of ankle |
| 484101000006114 | Ankylosis of the inferior radioulnar joint |
| 601231000006112 | Crushing injuries of thorax with abdomen, lower back and pelvis with limb(s) |
| 623381000006114 | Dislocations, sprains and strains involving multiple regions of upper limb(s) |
| 736171000006110 | Localised, secondary osteoarthritis of the pelvic region and thigh |
| 819861000006110 | Helminthiasis with arthropathy of the pelvic region and thigh |
| 1660221000006115 | Benign coital headache |
| 309788014 | Rheumatoid arthritis of spine |
| 363811000006111 | Infective polyarthritis |
| 789621000006119 | Hyperuricaemia without signs of inflammatory arthritis and tophaceous disease |
| 1786081000006116 | Benign cough headache |
| 1927491000006111 | Irritable bowel syndrome characterised by alternating bowel habit |
| 47011000006118 | Open wounds involving thorax with abdomen, lower back and pelvis |
| 54641000006115 | Osteoarthritis of wrist |
| 92681000006110 | Traumatic arthropathy of metacarpophalangeal joint |
| 130621000006112 | Sprain finger, distal interphalangeal joint, radial collateral ligament |
| 131011000006118 | Sprain thumb, interphalangeal joint, ulnar collateral ligament |
| 149911000006117 | Seropositive rheumatoid arthritis |
| 162141000006118 | Rheumatoid arthritis of sacroiliac joint |
| 219271000000117 | Seronegative arthritis |
| 221491000000111 | Osteoarthritis of elbow |
| 491821000006114 | Arthralgia of distal interphalangeal joint of finger |
| 492111000006116 | Arthritis of acromioclavicular joint |
| 299347013 | Drug induced headache |
| 299365012 | Inflammatory neuropathy |
| 309942011 | Localised osteoarthritis |
| 310011016 | Osteoarthritis of shoulder |
| 310205010 | Arthropathy of joint of hand |
| 311705018 | Neuralgia |
| 312495012 | Disorder of musculoskeletal system |
| 317435015 | Musculoskeletal chest pain |
| 325487016 | Dislocations, sprains and strains involving multiple body regions |
| 359848019 | Ankylosis of spine |
| 400285018 | Thoracic neuritis |
| 493251000006115 | Arthropathy with other viral disease, of forearm |
| 493331000006119 | Arthropathy with other viral disease, of upper arm |
| 889721000006113 | Post-viral arthropathy |
| 890971000006115 | Back disorders - other |
| 891731000006111 | Myalgia/myositis - shoulder |
| 891771000006114 | Neuralgia/neuritis -ankle/foot |
| 891811000006114 | Neuralgia/neuritis - fore arm |
| 894961000006119 | Musculoskeletal x-ray abn. [D] |
| 896331000006116 | Sprained knee NOS |
| 896361000006113 | Sprain - ankle NOS |
| 931891000006117 | Headache disorder |
| 932681000006110 | Other peripheral neuropathy |
| 933101000006116 | Neck injury |
| 989371000006119 | Osteoarthritis - knee joint |
| 990091000006111 | Sprained hip |
| 990111000006119 | Sprained knee |
| 473482013 | Sacroiliac sprain |
| 309464013 | Pyogenic arthritis NOS |
| 603591000006113 | Crystal arthropathy NOS, of PIP joint of finger |
| 297531019 | Hereditary or idiopathic peripheral neuropathy NOS |
| 310148010 | Unspecified polyarthropathy of unspecified site |
| 320316019 | Coccyx sprain |
| 461577011 | [Q] Lateral spinal stenosis |
| 312528016 | [X]Other specific arthropathies, not elsewhere classified |
| 461006012 | [V]Other specified psychological or physical strain |
| 461440017 | [X]Other physical and mental strain related to work |
| 130561000006119 | Sprain & strain of oth & unspecif parts of should girdle |
| 363821000006115 | [X]Arthropathies in other specified diseases CE |
| 405781000006116 | [X]Oth/unspecif sympt & signs involv nerv/musculosk systems |
| 427431000006110 | [X]Sprain & strain of oth & unspecif parts of should girdle |
| 424981000006114 | [X]Rheumatoid arthritis+involvement/other organs or systems |
| 492171000006113 | Arthritis associated with other disease, IP joint of toe |
| 492201000006112 | Arthritis associated with other disease, MCP joint |
| 492981000006114 | Arthropathy in Reiter's disease |
| 493191000006117 | Arthropathy with other bacterial disease, of other spec site |
| 497081000006112 | Atlanto-axial joint sprain |
| 538831000006115 | Carpal instability, ulnar translocation |
| 570651000006110 | Cls spinal # with incomplete thoracic cord lesion, T1-6 NOS |
| 603431000006110 | Crystal arthropathy NOS |
| 603451000006115 | Crystal arthropathy NOS, of 1st MTP joint |
| 709441000006113 | Fibromyalgia |
| 779421000006114 | Infective arthritis NOS, of MCP joint |
| 779591000006110 | Infective arthritis NOS, of tibio-fibular joint |
| 823951000006111 | Hip osteoarthitis NOS |
| 162231000006117 | Rheumatoid arthropathy + visceral/systemic involvement NOS |
| 211241000006115 | Postherpetic trigeminal neuralgia |
| 215061000006110 | Polyneuropathy in uraemia |
| 217001000006117 | Post-herpetic neuralgia |
| 265841000006115 | Oligoarticular osteoarthritis, unspec, of unspecified sites |
| 375981000006118 | [X]Crush inj of oth & unspecif parts of abdom/low back/pelv |
| 422991000006117 | [X]Polyneuropathy/systemic connective tissue disorders CE |
| 320141017 | Other elbow sprain |
| 320256019 | Knee sprain NOS |
| 320280015 | Foot sprain NOS |
| 320544019 | Other and ill-defined sprains and strains |
| 320546017 | Jaw sprain, unspecified |
| 320566014 | Sternum sprain NOS |
| 323927014 | Other face and neck injuries |
| 400197012 | Arthropathy NOS, of the pelvic region and thigh |
| 18181000006118 | Osteoarthritis of spine |
| 28831000006117 | Other infect/parasit dis with arthropathy of pelvic / thigh |
| 29261000006119 | Other infect/parasit dis with arthropathy of multiple sites |
| 84571000006115 | Type 2 diabetes mellitus with neuropathic arthropathy |
| 92731000006117 | Traumatic arthropathy of sacro-iliac joint |
| 131081000006113 | Sprain ulnar carpal complex non-specific |
| 299349011 | [X]Other disorders of trigeminal nerve |
| 309537014 | Arthropathy associated with mycoses, of the forearm |
| 309597011 | Infective arthritis NOS, of the upper arm |
| 309608017 | Infective arthritis NOS, of wrist |
| 309618010 | Infective arthritis NOS, of ankle |
| 309626019 | Infective arthritis NOS, of other specified site |
| 309685017 | Other crystal arthropathies |
| 309686016 | Other crystal arthropathies of unspecified site |
| 309692010 | Other crystal arthropathies of the lower leg |
| 309701018 | Crystal arthropathy NOS, of the upper arm |
| 309764012 | Arthritis associated with other disease, elbow |
| 309880013 | Generalised osteoarthritis NOS |
| 309953017 | Localised osteoarthritis, unspecified, of the hand |
| 309975015 | Oligoarticular osteoarthritis, unspecified, of upper arm |
| 310016014 | Osteoarthritis NOS, of wrist |
| 310034013 | Other and unspecified arthropathies |
| 310105016 | Climacteric arthritis NOS |
| 310106015 | Transient arthropathy of unspecified site |
| 310167014 | Unspecified monoarthritis of the shoulder region |
| 310169012 | Unspecified monoarthritis of the forearm |
| 310182016 | Other specified arthropathy of unspecified site |
| 310197018 | Arthropathy NOS, of unspecified site |
| 311704019 | Myalgia or myositis NOS |
| 311706017 | Neuralgia unspecified |
| 311768018 | Nonarticular rheumatism NOS |
| 312571013 | [X]Other instability of joint |
| 312630014 | [X]Other dorsalgia |
| 312800015 | Spinal meningocele of unspecified site |
| 82989010 | Closed dislocation of sacroiliac joint |
| 253116010 | Headache site NOS |
| 1165801000000110 | Rheumatology service home visit |
| 875201000006117 | Therap.asp.- musculoskelet.NOS |
| 889891000006116 | Osteoarthritis - wrist joint |
| 896281000006113 | Sprain - lower leg |
| 896411000006117 | Sprained neck |
| 240071000006115 | Patellofemoral osteoarthritis |
| 559621000000119 | Suspected inflammatory arthritis |
| 2695828012 | Gouty arthritis of toe |
| 2791650019 | Irritable bowel syndrome characterised by constipation |
| 1221279016 | Sacroiliac ankylosis |
| 1492276018 | Osteoarthritis of cervical spine |
| 1492281010 | Osteoarthritis of thoracic spine |
| 1494798013 | Sternum sprain |
| 1495246018 | Sprained toe |
| 1786065018 | Hereditary motor and sensory neuropathy type I |
| 2477021015 | Ochronotic arthropathy |
| 391096011 | Forearm sprain |
| 391105019 | Toe sprain |
| 393896016 | Intracranial destruction of trigeminal nerve (V) |
| 400247016 | Arthralgia of the upper arm |
| 455431014 | Prolapsed lumbar intervertebral disc with sciatica |
| 459740018 | Intractable breast pain |
| 473460018 | Charcot's arthropathy |
| 475273015 | Ulnar neuropathy |
| 295342013 | Psychogenic musculoskeletal symptoms |
| 297553017 | Polyneuropathy in disease EC |
| 299136013 | Ossicle ankylosis (excluding malleus) |
| 309552019 | Helminthiasis with arthropathy of the hand |
| 309750012 | Arthropathy associated with respiratory disorders |
| 309881012 | Localised, primary osteoarthritis |
| 309898012 | Localised, primary osteoarthritis of the ankle and foot |
| 309918012 | Localised, secondary osteoarthritis of the shoulder region |
| 310050017 | Traumatic arthropathy of the shoulder region |
| 310056011 | Traumatic arthropathy of the ankle and foot |
| 310068019 | Traumatic arthropathy-hip |
| 310070011 | Traumatic arthropathy-knee |
| 4765019 | Postherpetic neuralgia |
| 35988013 | Open wound of back with complication |
| 50702011 | Mononeuritis multiplex |
| 72981015 | Glossopharyngeal neuralgia |
| 84036012 | Palindromic rheumatism |
| 129260010 | Menopausal arthritis |
| 138248010 | Basilar migraine |
| 143436010 | Idiopathic peripheral autonomic neuropathy |
| 249890015 | FH: Migraine |
| 252314011 | C/O - low back pain |
| 253109014 | Bilateral headache |
| 253115014 | Parietal headache |
| 253118011 | Aching headache |
| 257897018 | Rheumatoid factor negative |
| 262959010 | Headache caused by oral contraceptive pill |
| 310072015 | Traumatic arthropathy-ankle |
| 310082019 | Allergic arthritis of the shoulder region |
| 310104017 | Climacteric arthritis of multiple sites |
| 311194016 | Idiopathic cervical spinal stenosis |
| 311195015 | Degenerative cervical spinal stenosis |
| 311211016 | Rheumatic torticollis |
| 311234013 | Iatrogenic thoracic spinal stenosis |
| 311256011 | Thoracic and lumbosacral neuritis |
| 311272015 | Sacroiliac disorder |
| 319763013 | Closed spinal dislocation with complete thoracic cord lesion |
| 320114010 | Coracohumeral sprain |
| 320159019 | Sprain ulnar-carpal meniscus |
| 320162016 | Sprain ulnar collateral ligament |
| 320242016 | Sprain or partial tear, knee, lateral collateral ligament |
| 320278014 | Sprain, flexor tendon, foot |
| 320545018 | Septal cartilage nose sprain |
| 323826018 | Closed injury sciatic nerve |
| 345355014 | Status migrainosus |
| 297569012 | Polyneuropathy in disease NOS |
| 309922019 | Localised, secondary osteoarthritis of the forearm |
| 309934010 | Localised, secondary osteoarthritis of other specified site |
| 320311012 | Sacral sprain, unspecified |
| 400181019 | Localised osteoarthritis, unspecified, of the lower leg |
| 493271000006113 | Arthropathy with other viral disease, of lower leg |
| 891761000006119 | Myalgia/myositis - NOS |
| 891831000006115 | Neuralgia/neuritis - shoulder |
| 896161000006110 | Dislocations/sprains NOS |
| 990351000006115 | Sprain - fore arm |
| 889851000006110 | Polyarthropathy NOS -inflammat |
| 257908018 | Rheumatoid factor NOS |
| 309849015 | Other specified inflammatory polyarthropathy NOS |
| 684221000006112 | Myalgic encephalomyelitis |
| 400222018 | Joint ankylosis of the upper arm |
| 889621000006117 | Arthritis/arthrosis |
| 415621000006114 | [X]Other specified injuries of abdomen, lower back & pelvis |
| 427421000006112 | [X]Sprain & strain of oth & unsp parts of lumb spine & pelv |
| 484171000006115 | Ankylosis/instability of cervical,thoracic or lumbar spine |
| 492731000006111 | Arthropathy associated with other conditions EC |
| 493151000006111 | Arthropathy with other bacterial disease, of forearm |
| 493171000006118 | Arthropathy with other bacterial disease, of lower leg |
| 497061000006119 | Atlanto-axial ankylosis |
| 523461000006116 | Bouchard's nodes with arthropathy |
| 567561000006119 | Closed spinal dislocation with cervical cord lesion, unspec |
| 567601000006119 | Closed spinal dislocation with lumbar cord lesion, unspec |
| 570631000006115 | Cls spinal # with incomplete cervical cord lesion, C1-4 NOS |
| 700211000006115 | Moebius' ophthalmoplegic migraine |
| 736711000006119 | Localised osteoarthritis, unspecified, of shoulder region |
| 162191000006110 | Rheumatoid arthritis of tibio-fibular joint |
| 211331000006117 | Postinfectious polyneuritis |
| 217671000006115 | Postdysenteric reactive arthropathy of multiple sites |
| 264471000006113 | Oligoarticular osteoarthritis, unspecified, of pelvis/thigh |
| 363801000006113 | [X]Arthritis in other infectious and parasitic diseases CE |
| 311035015 | Other specified arthropathies |
| 311240018 | Lumbar spinal stenosis secondary to other disease |
| 311271010 | Sacral instability NOS |
| 311746011 | Other musculoskeletal limb symptoms |
| 312527014 | [X]Other specified crystal arthropathies |
| 312529012 | [X]Other specified arthritis |
| 312792011 | Spinal hydromeningocele NOS |
| 320237012 | Thigh sprain NOS |
| 320251012 | Other specified knee sprain |
| 320252017 | Other specified leg sprain |
| 320270019 | Ankle sprain NOS |
| 320271015 | Foot sprain, unspecified |
| 320313010 | Sacrum sprain NOS |
| 320576012 | Sprain of pelvis NOS |
| 320578013 | Other specified sprains and strains |
| 400165012 | Arthropathy in Behcet's syndrome of unspecified site |
| 400198019 | Arthropathy NOS, of the lower leg |
| 400223011 | Joint ankylosis of the forearm |
| 460303019 | [V]Personal history of arthritis |
| 28241000006111 | Other infect/parasit dis with arthropathy of shoulder region |
| 34231000006110 | Other congenital musculoskeletal deformity |
| 297351016 | Migraine variant NOS |
| 297356014 | Migraine NOS |
| 297494010 | Mononeuritis upper limb NOS |
| 297505016 | Other mononeuritis lower limb |
| 297506015 | Unspecified mononeuritis lower limb |
| 297545013 | Polyneuropathy in collagen vascular disease NOS |
| 309551014 | Helminthiasis with arthropathy of the forearm |
| 309604015 | Infective arthritis NOS, of sternoclavicular joint |
| 309675018 | Gouty arthritis of the upper arm |
| 309676017 | Gouty arthritis of the forearm |
| 309697016 | Other crystal arthropathy NOS |
| 309771019 | Arthritis associated with other disease, hip |
| 309896011 | Localised, primary osteoarthritis of the lower leg |
| 310081014 | Allergic arthritis of unspecified site |
| 310083012 | Allergic arthritis of the upper arm |
| 310098014 | Climacteric arthritis of the forearm |
| 310156013 | Unspecified polyarthropathy of other specified site |
| 310172017 | Unspecified monoarthritis of the lower leg |
| 310181011 | Other specified arthropathy |
| 310217012 | Arthropathy NOS, of multiple sites |
| 310444016 | Carpal instability, other |
| 310557014 | Ankylosis of joint NOS |
| 317142014 | [D]Other nervous and musculoskeletal symptoms |
| 2129721000000119 | Management of irritable bowel syndrome |
| 1756651000000117 | Frequent episodic tension-type headache |
| 889861000006112 | Osteoarthritis -multiple joint |
| 890391000006115 | Ankylosis - shoulder joint |
| 895331000006114 | #Thoracic spine + cord lesion |
| 896311000006110 | Sprain - medial knee ligament |
| 693461000006115 | Musculoskeletal and connective tissue diseases |
| 495040011 | Pyogenic arthritis |
| 502690012 | Pauciarticular onset juvenile chronic arthritis |
| 504101018 | Polyneuropathy in beriberi |
| 508047017 | Osteoarthritis of spine |
| 1221607010 | Hip sprain |
| 1228298012 | Arthralgia of multiple joints |
| 399416017 | Inflammatory and toxic neuropathy |
| 400245012 | Arthralgia of the shoulder region |
| 400276018 | Brachial (cervical) neuritis |
| 400282015 | Spinal stenosis, excluding cervical region |
| 402914014 | Sprain of knee and leg |
| 416139010 | Low back pain |
| 459296014 | Type 1 diabetes mellitus with neuropathic arthropathy |
| 484410014 | Sacroiliac instability |
| 297337016 | Migraine variants |
| 297543018 | Polyneuropathy in polyarteritis nodosa |
| 251795011 | H/O: osteoarthritis |
| 252573011 | Abdominal migraine - symptom |
| 253105015 | Generalised headache |
| 257896010 | Rheumatoid factor positive |
| 7278014 | Arthritis |
| 19796013 | Hereditary sensory neuropathy |
| 30978014 | Lumbar spinal stenosis |
| 38727013 | Sciatica |
| 74641011 | Gonococcal arthritis |
| 126030010 | Abdominal migraine |
| 136713011 | Chronic pain |
| 158058014 | Intercostal myalgia |
| 309489019 | Arthropathy in Behcet's syndrome of the ankle and foot |
| 309563016 | Reactive arthropathy of acromioclavicular joint |
| 309674019 | Gouty arthritis of the shoulder region |
| 309743013 | Arthropathy in ulcerative colitis |
| 309789018 | Rheumatoid arthritis of shoulder |
| 309798015 | Rheumatoid arthritis of hip |
| 309800010 | Rheumatoid arthritis of knee |
| 309833017 | Juvenile arthritis in Crohn's disease |
| 310059016 | Traumatic arthropathy of shoulder |
| 310064017 | Traumatic arthropathy-wrist |
| 310121014 | Transient arthropathy-elbow |
| 310137015 | Transient arthropathy of subtalar joint |
| 311113014 | Enterobacterial spondylitis |
| 311281014 | Atlanto-occipital ankylosis |
| 311283012 | Cervical spine ankylosis |
| 311681019 | Spasm of back muscles |
| 319779014 | Open spinal dislocation with complete thoracic cord lesion |
| 319837010 | Closed spinal subluxation with anterior cervical cord lesion |
| 319870017 | Closed spinal subluxation with complete lumbar cord lesion |
| 320136013 | Sprain, elbow joint, lateral collateral ligament |
| 320138014 | Sprain, elbow joint, ulnar collateral ligament |
| 320160012 | Sprain triangular fibrocartilage |
| 320177012 | Carpometacarpal sprain |
| 320179010 | Interphalangeal sprain |
| 320214018 | Sprain, flexor pollicis longus tendon |
| 320215017 | Sprain, extensor pollicis longus tendon |
| 320216016 | Sprain tendon of finger |
| 320227019 | Iliofemoral sprain |
| 320310013 | Sacrum sprain |
| 320569019 | Sprain, symphysis pubis |
| 345594014 | Axonal sensorimotor neuropathy |
| 484753013 | Arthralgia of knee |
| 256125011 | O/E - musculoskeletal |
| 299359012 | Mononeuropathy of upper limb |
| 310027011 | Osteoarthritis of subtalar joint |
| 317145011 | Musculoskeletal pain |
| 359389010 | Osteoarthritis of finger |
| 391102016 | Sprain of joint |
| 399415018 | Mononeuropathy |
| 402912013 | Sprain of hand |
| 65811000006110 | Vascular headache |
| 92621000006111 | Traumatic arthropathy of 1st metatarsophalangeal joint |
| 130581000006112 | Late effect of sprain AND/OR strain without tendon injury |
| 130661000006118 | Sprain finger, proximal interphalangeal joint, radial collateral ligament |
| 130681000006111 | Sprain of metacarpophalangeal joint |
| 131001000006116 | Sprain thumb, interphalangeal joint, radial collateral ligament |
| 131351000006115 | Strain of hamstring tendon |
| 131361000006118 | Sprain of hip joint |
| 131421000006118 | Strain of long head of biceps |
| 162041000006117 | Rheumatoid arthritis of distal interphalangeal joint of finger |
| 162131000006111 | Rheumatoid arthritis of proximal interphalangeal joint of finger |
| 212551000006110 | Post-zoster neuralgia |
| 215271000000119 | Neck pain |
| 399821000006115 | Neuropathy |
| 567641000006117 | Closed spinal dislocation with posterior thoracic cord lesion |
| 567711000006112 | Closed spinal subluxation with posterior cervical cord lesion |
| 750721000006113 | Late effect of musculoskeletal and connective tissue injuries |
| 757841000006114 | Ankylosis of joint of hand |
| 775571000006111 | Injury of muscle and tendon of abdomen, lower back and pelvis |
| 780181000006118 | Infective arthritis |
| 309720016 | Crystal arthropathy of knee |
| 377491000006117 | [X]Dislocation, sprain and strain of unspecified joint and ligament of trunk |
| 409041000006110 | [X]Other disorders of the musculoskeletal system and connective tissue |
