# Supplementary material for: Trends of long-term opioid therapy and subsequent discontinuation among people with chronic non-cancer pain in UK primary care: A retrospective cohort study
Source: PLoS One. 2025 Jun 26;20(6):e0326604. doi: 10.1371/journal.pone.0326604 (PMC12200650; doi:10.1371/journal.pone.0326604)
Supplement: S3 Table — (DOCX) [file pone.0326604.s006.docx]

# **S3 Table. Opioid product code list**

| **prodcodeid** | **Product name** |
| --- | --- |
| 1029741000033111 | Paracetamol & Codeine Dispersible tablets |
| 3343341000033117 | Buprenorphine 5micrograms/hour transdermal patches |
| 11029341000033114 | Butec 5micrograms/hour transdermal patches (Qdem Pharmaceuticals Ltd) |
| 13751341000033119 | Rebrikel 5micrograms/hour transdermal patches (Zentiva) |
| 12603441000033118 | Bunov 5micrograms/hour transdermal patches (Glenmark Pharmaceuticals Europe Ltd) |
| 12325741000033112 | Bupramyl 5micrograms/hour transdermal patches (Mylan) |
| 11756241000033110 | Sevodyne 5micrograms/hour transdermal patches (Aspire Pharma Ltd) |
| 11732541000033112 | Reletrans 5micrograms/hour transdermal patches (Sandoz Ltd) |
| 3343641000033113 | BuTrans 5micrograms/hour transdermal patches (Napp Pharmaceuticals Ltd) |
| 11730941000033119 | Panitaz 5micrograms/hour transdermal patches (Dr Reddy's Laboratories (UK) Ltd) |
| 12409541000033116 | Busiete 5micrograms/hour transdermal patches (Teva UK Ltd) |
| 10598241000033114 | Yemex 12micrograms/hour transdermal patches (Sandoz Ltd) |
| 4022641000033116 | Matrifen 12micrograms/hour transdermal patches (Teva UK Ltd) |
| 6440741000033114 | Fencino 12micrograms/hour transdermal patches (Ethypharm UK Ltd) |
| 11507641000033116 | Victanyl 12micrograms/hour transdermal patches (Accord Healthcare Ltd) |
| 3839341000033117 | Fentanyl 12micrograms/hour transdermal patches |
| 8884441000033117 | Opiodur 12micrograms/hour transdermal patches (Zentiva) |
| 3839441000033111 | Durogesic DTrans 12micrograms/hour transdermal patches (Janssen-Cilag Ltd) |
| 4386641000033113 | Mezolar Matrix 12micrograms/hour transdermal patches (Sandoz Ltd) |
| 9204341000033115 | Mylafent 12micrograms/hour transdermal patches (Mylan) |
| 5300441000033116 | Osmanil 12micrograms/hour transdermal patches (Zentiva) |
| 12106841000033113 | Butec 15micrograms/hour transdermal patches (Qdem Pharmaceuticals Ltd) |
| 13717441000033113 | Sevodyne 15micrograms/hour transdermal patches (Aspire Pharma Ltd) |
| 11077241000033112 | BuTrans 15micrograms/hour transdermal patches (Napp Pharmaceuticals Ltd) |
| 11732341000033117 | Reletrans 15micrograms/hour transdermal patches (Sandoz Ltd) |
| 11077041000033116 | Buprenorphine 15micrograms/hour transdermal patches |
| 12409641000033115 | Busiete 10micrograms/hour transdermal patches (Teva UK Ltd) |
| 11756341000033117 | Sevodyne 10micrograms/hour transdermal patches (Aspire Pharma Ltd) |
| 11732241000033110 | Reletrans 10micrograms/hour transdermal patches (Sandoz Ltd) |
| 3343741000033116 | BuTrans 10micrograms/hour transdermal patches (Napp Pharmaceuticals Ltd) |
| 3343441000033111 | Buprenorphine 10micrograms/hour transdermal patches |
| 11730741000033117 | Panitaz 10micrograms/hour transdermal patches (Dr Reddy's Laboratories (UK) Ltd) |
| 12603241000033119 | Bunov 10micrograms/hour transdermal patches (Glenmark Pharmaceuticals Europe Ltd) |
| 12325941000033110 | Bupramyl 10micrograms/hour transdermal patches (Mylan) |
| 11029441000033115 | Butec 10micrograms/hour transdermal patches (Qdem Pharmaceuticals Ltd) |
| 575041000033113 | Fentanyl 25micrograms/hour transdermal patches |
| 10336841000033116 | Mylafent 25micrograms/hour transdermal patches (Mylan) |
| 5007441000033116 | Victanyl 25micrograms/hour transdermal patches (Accord Healthcare Ltd) |
| 3333041000033118 | Tilofyl 25micrograms/hour transdermal patches (Tillomed Laboratories Ltd) |
| 4022741000033113 | Matrifen 25micrograms/hour transdermal patches (Teva UK Ltd) |
| 4502841000033111 | Osmach 25micrograms/hour transdermal patches (Teva UK Ltd) |
| 6440841000033116 | Fencino 25micrograms/hour transdermal patches (Ethypharm UK Ltd) |
| 8884541000033116 | Opiodur 25micrograms/hour transdermal patches (Zentiva) |
| 4956041000033118 | Osmanil 25micrograms/hour transdermal patches (Zentiva) |
| 4426341000033119 | Fentalis Reservoir 25micrograms/hour transdermal patches (Sandoz Ltd) |
| 490341000033116 | Durogesic 25micrograms transdermal patches (Janssen-Cilag Ltd) |
| 4387041000033117 | Mezolar Matrix 25micrograms/hour transdermal patches (Sandoz Ltd) |
| 3248041000033118 | Durogesic DTrans 25micrograms/hour transdermal patches (Janssen-Cilag Ltd) |
| 10598141000033119 | Yemex 25micrograms/hour transdermal patches (Sandoz Ltd) |
| 12326041000033117 | Bupramyl 20micrograms/hour transdermal patches (Mylan) |
| 12409741000033112 | Busiete 20micrograms/hour transdermal patches (Teva UK Ltd) |
| 12603341000033112 | Bunov 20micrograms/hour transdermal patches (Glenmark Pharmaceuticals Europe Ltd) |
| 11029541000033119 | Butec 20micrograms/hour transdermal patches (Qdem Pharmaceuticals Ltd) |
| 3343841000033114 | BuTrans 20micrograms/hour transdermal patches (Napp Pharmaceuticals Ltd) |
| 11730841000033110 | Panitaz 20micrograms/hour transdermal patches (Dr Reddy's Laboratories (UK) Ltd) |
| 11756441000033111 | Sevodyne 20micrograms/hour transdermal patches (Aspire Pharma Ltd) |
| 11732441000033111 | Reletrans 20micrograms/hour transdermal patches (Sandoz Ltd) |
| 3343541000033112 | Buprenorphine 20micrograms/hour transdermal patches |
| 2737541000033111 | Buprenorphine 35micrograms/hour transdermal patches |
| 12194941000033112 | Relevtec 35micrograms/hour transdermal patches (Sandoz Ltd) |
| 12428441000033117 | Turgeon 35micrograms/hour transdermal patches (Teva UK Ltd) |
| 11484341000033112 | Bupeaze 35micrograms/hour transdermal patches (Dr Reddy's Laboratories (UK) Ltd) |
| 8882741000033116 | Hapoctasin 35micrograms/hour transdermal patches (Accord Healthcare Ltd) |
| 11780641000033117 | Buplast 35micrograms/hour transdermal patches (Mylan) |
| 11507941000033111 | Prenotrix 35micrograms/hour transdermal patches (Genesis Pharmaceuticals Ltd) |
| 12603541000033117 | Carlosafine 35micrograms/hour transdermal patches (Glenmark Pharmaceuticals Europe Ltd) |
| 2737841000033113 | Transtec 35micrograms/hour transdermal patches (Napp Pharmaceuticals Ltd) |
| 8962341000033111 | Mezolar Matrix 37.5microgram/hour transdermal patches (Sandoz Ltd) |
| 8962241000033118 | Fentanyl 37.5microgram/hour transdermal patches |
| 11469241000033116 | Fentanyl 40micrograms/dose transdermal system |
| 12603641000033116 | Carlosafine 52.5micrograms/hour transdermal patches (Glenmark Pharmaceuticals Europe Ltd) |
| 11484441000033118 | Bupeaze 52.5micrograms/hour transdermal patches (Dr Reddy's Laboratories (UK) Ltd) |
| 8882841000033114 | Hapoctasin 52.5micrograms/hour transdermal patches (Accord Healthcare Ltd) |
| 2737941000033117 | Transtec 52.5micrograms/hour transdermal patches (Napp Pharmaceuticals Ltd) |
| 2737641000033112 | Buprenorphine 52.5micrograms/hour transdermal patches |
| 11780741000033114 | Buplast 52.5micrograms/hour transdermal patches (Mylan) |
| 11577141000033119 | Prenotrix 52.5micrograms/hour transdermal patches (Genesis Pharmaceuticals Ltd) |
| 12428541000033116 | Turgeon 52.5micrograms/hour transdermal patches (Teva UK Ltd) |
| 12195041000033112 | Relevtec 52.5micrograms/hour transdermal patches (Sandoz Ltd) |
| 5007541000033115 | Victanyl 50micrograms/hour transdermal patches (Accord Healthcare Ltd) |
| 10598041000033118 | Yemex 50micrograms/hour transdermal patches (Sandoz Ltd) |
| 3248141000033119 | Durogesic DTrans 50micrograms/hour transdermal patches (Janssen-Cilag Ltd) |
| 6440941000033112 | Fencino 50micrograms/hour transdermal patches (Ethypharm UK Ltd) |
| 490441000033110 | Durogesic 50micrograms transdermal patches (Janssen-Cilag Ltd) |
| 3332941000033111 | Tilofyl 50micrograms/hour transdermal patches (Tillomed Laboratories Ltd) |
| 4022841000033115 | Matrifen 50micrograms/hour transdermal patches (Teva UK Ltd) |
| 8884641000033115 | Opiodur 50micrograms/hour transdermal patches (Zentiva) |
| 4426241000033112 | Fentalis Reservoir 50micrograms/hour transdermal patches (Sandoz Ltd) |
| 575141000033112 | Fentanyl 50micrograms/hour transdermal patches |
| 4386741000033116 | Mezolar Matrix 50micrograms/hour transdermal patches (Sandoz Ltd) |
| 9204441000033114 | Mylafent 50micrograms/hour transdermal patches (Mylan) |
| 4956141000033119 | Osmanil 50micrograms/hour transdermal patches (Zentiva) |
| 4502941000033115 | Osmach 50micrograms/hour transdermal patches (Teva UK Ltd) |
| 12356141000033117 | Co-codamol 60mg/1000mg tablets |
| 1741241000033116 | Kapake Insts 60mg/1000mg effervescent powder sachets (Galen Ltd) |
| 4386841000033114 | Mezolar Matrix 75micrograms/hour transdermal patches (Sandoz Ltd) |
| 3248241000033114 | Durogesic DTrans 75micrograms/hour transdermal patches (Janssen-Cilag Ltd) |
| 4956241000033114 | Osmanil 75micrograms/hour transdermal patches (Zentiva) |
| 4426141000033117 | Fentalis Reservoir 75micrograms/hour transdermal patches (Sandoz Ltd) |
| 575241000033117 | Fentanyl 75micrograms/hour transdermal patches |
| 10597941000033116 | Yemex 75micrograms/hour transdermal patches (Sandoz Ltd) |
| 8884741000033112 | Opiodur 75micrograms/hour transdermal patches (Zentiva) |
| 4503041000033113 | Osmach 75micrograms/hour transdermal patches (Teva UK Ltd) |
| 5007241000033117 | Victanyl 75micrograms/hour transdermal patches (Accord Healthcare Ltd) |
| 4022941000033111 | Matrifen 75micrograms/hour transdermal patches (Teva UK Ltd) |
| 6441041000033119 | Fencino 75micrograms/hour transdermal patches (Ethypharm UK Ltd) |
| 490541000033111 | Durogesic 75micrograms transdermal patches (Janssen-Cilag Ltd) |
| 3332841000033115 | Tilofyl 75micrograms/hour transdermal patches (Tillomed Laboratories Ltd) |
| 9204541000033110 | Mylafent 75micrograms/hour transdermal patches (Mylan) |
| 11780841000033116 | Buplast 70micrograms/hour transdermal patches (Mylan) |
| 11577241000033114 | Prenotrix 70micrograms/hour transdermal patches (Genesis Pharmaceuticals Ltd) |
| 12603741000033113 | Carlosafine 70micrograms/hour transdermal patches (Glenmark Pharmaceuticals Europe Ltd) |
| 2737741000033115 | Buprenorphine 70micrograms/hour transdermal patches |
| 12428641000033115 | Turgeon 70micrograms/hour transdermal patches (Teva UK Ltd) |
| 12195141000033111 | Relevtec 70micrograms/hour transdermal patches (Sandoz Ltd) |
| 11484541000033117 | Bupeaze 70micrograms/hour transdermal patches (Dr Reddy's Laboratories (UK) Ltd) |
| 2738041000033119 | Transtec 70micrograms/hour transdermal patches (Napp Pharmaceuticals Ltd) |
| 13754841000033114 | Morphine sulfate 500micrograms/5ml oral solution |
| 5007341000033110 | Victanyl 100micrograms/hour transdermal patches (Accord Healthcare Ltd) |
| 574941000033113 | Fentanyl 100micrograms/hour transdermal patches |
| 490241000033114 | Durogesic 100micrograms transdermal patches (Janssen-Cilag Ltd) |
| 4956341000033116 | Osmanil 100micrograms/hour transdermal patches (Zentiva) |
| 4426041000033116 | Fentalis Reservoir 100micrograms/hour transdermal patches (Sandoz Ltd) |
| 4503141000033112 | Osmach 100micrograms/hour transdermal patches (Ratiopharm UK Ltd) |
| 8884341000033111 | Opiodur 100micrograms/hour transdermal patches (Zentiva) |
| 3333141000033119 | Tilofyl 100micrograms/hour transdermal patches (Tillomed Laboratories Ltd) |
| 6441141000033115 | Fencino 100micrograms/hour transdermal patches (Ethypharm UK Ltd) |
| 9204641000033111 | Mylafent 100micrograms/hour transdermal patches (Mylan) |
| 4386941000033118 | Mezolar Matrix 100micrograms/hour transdermal patches (Sandoz Ltd) |
| 10597841000033112 | Yemex 100micrograms/hour transdermal patches (Sandoz Ltd) |
| 4023041000033118 | Matrifen 100micrograms/hour transdermal patches (Teva UK Ltd) |
| 3248341000033116 | Durogesic DTrans 100micrograms/hour transdermal patches (Janssen-Cilag Ltd) |
| 625141000033115 | Galcodine 3mg/5ml linctus paediatric (Thornton & Ross Ltd) |
| 336041000033115 | Codeine 3mg/5ml linctus paediatric |
| 931041000033118 | Morphine Hydrochloride Mixture 916 micrograms |
| 728641000033114 | Hydromorphone 1.3mg capsules |
| 1029141000033112 | Palladone 1.3mg capsules (Napp Pharmaceuticals Ltd) |
| 1564541000033112 | Zydol SR 100mg tablets (Grunenthal Ltd) |
| 12350041000033118 | Tilodol SR 100mg tablets (Sandoz Ltd) |
| 4417741000033112 | Zeridame SR 100mg tablets (Actavis UK Ltd) |
| 1549241000033116 | Zamadol SR 100mg capsules (Mylan) |
| 930741000033113 | Morcap SR 100mg capsules (Hospira UK Ltd) |
| 1462641000033113 | Tramadol 100mg modified-release capsules |
| 930041000033110 | Morphine 100mg modified-release granules sachets sugar free |
| 1702141000033113 | Tramadol 100mg effervescent powder sachets sugar free |
| 2753641000033115 | Filnarine SR 100mg tablets (Teva UK Ltd) |
| 2078341000033117 | Dromadol SR 100mg tablets (Teva UK Ltd) |
| 12353641000033115 | Zytram SR 100mg tablets (Qdem Pharmaceuticals Ltd) |
| 6133941000033117 | Palexia SR 100mg tablets (Grunenthal Ltd) |
| 2180641000033111 | Morphine Sulfate Suppositories 100 mg |
| 2912441000033113 | Morphgesic SR 100mg tablets (Advanz Pharma) |
| 1924241000033110 | Morphine 100mg modified-release capsules |
| 940141000033110 | MST Continus Suspension 100mg granules sachets (Napp Pharmaceuticals Ltd) |
| 3996441000033116 | Tradorec XL 100mg tablets (Endo Ventures Ltd) |
| 12346941000033118 | Oldaram 100mg modified-release tablets (Ranbaxy (UK) Ltd) |
| 4824241000033110 | Marol 100mg modified-release tablets (Teva UK Ltd) |
| 4523141000033117 | Tramquel SR 100mg capsules (Mylan) |
| 3909541000033113 | Mabron 100mg modified-release tablets (Teva UK Ltd) |
| 1014041000033118 | Oramorph Sr M/R tablets 100 mg |
| 1701941000033115 | Tramake Insts 100mg sachets (Galen Ltd) |
| 4028341000033114 | Larapam SR 100mg tablets (Sandoz Ltd) |
| 4459341000033112 | Tramulief SR 100mg tablets (Advanz Pharma) |
| 941041000033119 | MST Continus 100mg tablets (Napp Pharmaceuticals Ltd) |
| 6133241000033114 | Tapentadol 100mg modified-release tablets |
| 11567841000033116 | Maneo 100mg modified-release tablets (Mylan) |
| 12346441000033111 | Invodol SR 100mg tablets (Ennogen Healthcare Ltd) |
| 936541000033119 | Morphine 100mg modified-release tablets |
| 1462341000033117 | Tramadol 100mg modified-release tablets |
| 1561241000033112 | Zomorph 100mg modified-release capsules (Ethypharm UK Ltd) |
| 4899241000033117 | Maxitram SR 100mg capsules (Chiesi Ltd) |
| 6389141000033118 | Tramadol 100mg/ml oral drops |
| 1981941000033112 | Oxycodone 10mg modified-release tablets |
| 2912141000033117 | Morphgesic SR 10mg tablets (Advanz Pharma) |
| 929741000033117 | Morphine 10mg modified-release tablets |
| 10333241000033114 | Oxeltra 10mg modified-release tablets (Wockhardt UK Ltd) |
| 1561141000033117 | Zomorph 10mg modified-release capsules (Ethypharm UK Ltd) |
| 430341000033110 | Dextromoramide 10mg tablets |
| 373141000033110 | Co-dydramol 10mg/500mg tablets |
| 3179641000033116 | Dipipanone 10mg / Cyclizine 30mg tablets |
| 470841000033115 | Diconal tablets (Amdipharm Plc) |
| 428241000033118 | Dextromoramide Suppositories 10 mg |
| 12666341000033119 | Ixyldone 10mg modified-release tablets (Morningside Healthcare Ltd) |
| 7886341000033112 | Longtec 10mg modified-release tablets (Qdem Pharmaceuticals Ltd) |
| 1041241000033118 | Palfium 10mg tablets (Roche Products Ltd) |
| 10984941000033117 | Carexil 10mg modified-release tablets (Sandoz Ltd) |
| 1987241000033111 | Oxycodone 10mg capsules |
| 11808041000033113 | Leveraxo 10mg modified-release tablets (Mylan) |
| 1013941000033115 | Oramorph Sr M/R tablets 10 mg |
| 12347241000033113 | Eroset 500mg/10mg tablets (M & A Pharmachem Ltd) |
| 12636541000033115 | Renocontin 10mg modified-release tablets (Glenmark Pharmaceuticals Europe Ltd) |
| 8537441000033117 | Lynlor 10mg capsules (Accord Healthcare Ltd) |
| 936441000033115 | Morphine 10mg tablets |
| 1923941000033116 | Morphine 10mg modified-release capsules |
| 13708541000033112 | Oxycodone 10mg tablets |
| 940941000033112 | MST Continus 10mg tablets (Napp Pharmaceuticals Ltd) |
| 1403141000033111 | Syndol caplets (Sanofi) |
| 9177341000033118 | Reltebon 10mg modified-release tablets (Accord Healthcare Ltd) |
| 1278541000033116 | Sevredol 10mg tablets (Napp Pharmaceuticals Ltd) |
| 12664541000033119 | Oxypro 10mg modified-release tablets (Ridge Pharma Ltd) |
| 1273741000033117 | Sevredol Suppositories 10 mg |
| 10641541000033118 | Abtard 10mg modified-release tablets (Ethypharm UK Ltd) |
| 1987641000033114 | OxyContin 10mg modified-release tablets (Napp Pharmaceuticals Ltd) |
| 1988141000033117 | OxyNorm 10mg capsules (Napp Pharmaceuticals Ltd) |
| 1039441000033119 | Palfium Suppositories 10 mg |
| 1041941000033110 | Papaveretum Tablets 10 mg |
| 2753341000033111 | Filnarine SR 10mg tablets (Teva UK Ltd) |
| 9061641000033115 | Shortec 10mg capsules (Qdem Pharmaceuticals Ltd) |
| 12185341000033113 | Onexila XL 10mg tablets (Aspire Pharma Ltd) |
| 8048141000033110 | Oxylan 10mg modified-release tablets (Healthcare Pharma Ltd) |
| 934141000033117 | Morphine sulfate 10mg suppositories |
| 11243241000033114 | Zomestine 10mg modified-release tablets (Accord Healthcare Ltd) |
| 1140541000033115 | Propain caplets (Ceuta Healthcare Ltd) |
| 4898341000033115 | Targinact 20mg/10mg modified-release tablets (Napp Pharmaceuticals Ltd) |
| 4898241000033113 | Oxycodone 20mg / Naloxone 10mg modified-release tablets |
| 1988341000033119 | OxyNorm 10mg/ml concentrate oral solution (Napp Pharmaceuticals Ltd) |
| 12187141000033118 | Shortec 10mg/ml concentrate oral solution (Qdem Pharmaceuticals Ltd) |
| 13118741000033113 | Morphine (Opium tincture) 10mg/ml oral drops sugar free |
| 1987541000033113 | Oxycodone 10mg/ml oral solution sugar free |
| 2968841000033110 | Panadol Ultra 12.8mg/500mg tablets (GlaxoSmithKline Consumer Healthcare) |
| 2968641000033114 | Solpaflex tablets (GlaxoSmithKline Consumer Healthcare) |
| 2228341000033113 | Nurofen Plus tablets (Reckitt Benckiser Healthcare (UK) Ltd) |
| 12708841000033116 | Ibuprofen 200mg / Codeine 12.8mg tablets |
| 3077941000033110 | Cuprofen PLUS tablets (SSL International Plc) |
| 12583641000033114 | Solpadeine Max soluble tablets (Omega Pharma Ltd) |
| 5334541000033117 | Co-codamol 12.8mg/500mg tablets |
| 9292941000033111 | Solpadeine Max 12.8mg/500mg tablets (Omega Pharma Ltd) |
| 433441000033114 | DHC Continus 120mg tablets (Napp Pharmaceuticals Ltd) |
| 10492241000033118 | Longtec 120mg modified-release tablets (Qdem Pharmaceuticals Ltd) |
| 2068541000033117 | Morphine 120mg modified-release capsules |
| 6125341000033115 | Oxycodone 120mg modified-release tablets |
| 469441000033116 | Dihydrocodeine 120mg modified-release tablets |
| 944041000033117 | MXL 120mg capsules (Napp Pharmaceuticals Ltd) |
| 6125741000033119 | OxyContin 120mg modified-release tablets (Napp Pharmaceuticals Ltd) |
| 2068641000033116 | Morphine 150mg modified-release capsules |
| 4417641000033115 | Zeridame SR 150mg tablets (Actavis UK Ltd) |
| 4028541000033119 | Larapam SR 150mg tablets (Sandoz Ltd) |
| 4523241000033112 | Tramquel SR 150mg capsules (Mylan) |
| 1462441000033111 | Tramadol 150mg modified-release tablets |
| 4459441000033118 | Tramulief SR 150mg tablets (Advanz Pharma) |
| 12353741000033112 | Zytram SR 150mg tablets (Qdem Pharmaceuticals Ltd) |
| 6134041000033115 | Palexia SR 150mg tablets (Grunenthal Ltd) |
| 944141000033118 | MXL 150mg capsules (Napp Pharmaceuticals Ltd) |
| 1850141000033116 | Zydol XL 150mg tablets (Grunenthal Ltd) |
| 4824341000033117 | Marol 150mg modified-release tablets (Teva UK Ltd) |
| 6133341000033116 | Tapentadol 150mg modified-release tablets |
| 11568141000033114 | Maneo 150mg modified-release tablets (Mylan) |
| 12346641000033113 | Invodol SR 150mg tablets (Ennogen Healthcare Ltd) |
| 3909641000033114 | Mabron 150mg modified-release tablets (Teva UK Ltd) |
| 12350141000033119 | Tilodol SR 150mg tablets (Sandoz Ltd) |
| 1462741000033116 | Tramadol 150mg modified-release capsules |
| 3344141000033117 | Zamadol 24hr 150mg modified-release tablets (Mylan) |
| 1564641000033113 | Zydol SR 150mg tablets (Grunenthal Ltd) |
| 2183441000033116 | Dromadol XL 150mg tablets (IVAX Pharmaceuticals UK Ltd) |
| 1549341000033114 | Zamadol SR 150mg capsules (Mylan) |
| 2078441000033111 | Dromadol SR 150mg tablets (Teva UK Ltd) |
| 4899341000033110 | Maxitram SR 150mg capsules (Chiesi Ltd) |
| 10333341000033116 | Oxeltra 15mg modified-release tablets (Wockhardt UK Ltd) |
| 12636741000033111 | Renocontin 15mg modified-release tablets (Glenmark Pharmaceuticals Europe Ltd) |
| 6125441000033114 | OxyContin 15mg modified-release tablets (Napp Pharmaceuticals Ltd) |
| 6125041000033117 | Oxycodone 15mg modified-release tablets |
| 10491541000033114 | Longtec 15mg modified-release tablets (Qdem Pharmaceuticals Ltd) |
| 929941000033119 | Morphine 15mg modified-release tablets |
| 934641000033110 | Morphine hydrochloride 15mg suppositories |
| 12664641000033118 | Oxypro 15mg modified-release tablets (Ridge Pharma Ltd) |
| 940041000033111 | MST Continus 15mg tablets (Napp Pharmaceuticals Ltd) |
| 6431341000033116 | Co-codamol 15mg/500mg capsules |
| 9809641000033116 | Reltebon 15mg modified-release tablets (Accord Healthcare Ltd) |
| 933841000033114 | Morphine sulfate 15mg suppositories |
| 371141000033111 | Codeine 15mg tablets |
| 10641641000033117 | Abtard 15mg modified-release tablets (Ethypharm UK Ltd) |
| 6431441000033110 | Codipar 15mg/500mg capsules (Advanz Pharma) |
| 2850041000033116 | Codipar 15mg/500mg tablets (Advanz Pharma) |
| 2875341000033110 | Co-codamol 15mg/500mg tablets |
| 6137941000033114 | Kapake 15mg/500mg tablets (Galen Ltd) |
| 6386441000033115 | Co-codamol 15mg/500mg effervescent tablets sugar free |
| 6386541000033119 | Codipar 15mg/500mg effervescent tablets (Advanz Pharma) |
| 1036641000033110 | Palladone SR 16mg capsules (Napp Pharmaceuticals Ltd) |
| 738241000033114 | Hydromorphone 16mg modified-release capsules |
| 2093241000033114 | Morphine sulfate powder |
| 2086141000033111 | Codeine phosphate powder |
| 2093141000033119 | Morphine hydrochloride powder |
| 4434241000033119 | Morphine sulfate 5mg/5ml oral solution |
| 1987441000033112 | Oxycodone 5mg/5ml oral solution sugar free |
| 11444741000033116 | Morphine 0.1% in Intrasite gel |
| 12187041000033117 | Shortec liquid 1mg/ml oral solution (Qdem Pharmaceuticals Ltd) |
| 1988441000033113 | OxyNorm liquid 1mg/ml oral solution (Napp Pharmaceuticals Ltd) |
| 5234441000033111 | Targinact 5mg/2.5mg modified-release tablets (Napp Pharmaceuticals Ltd) |
| 5234241000033110 | Oxycodone 5mg / Naloxone 2.5mg modified-release tablets |
| 1029241000033117 | Palladone 2.6mg capsules (Napp Pharmaceuticals Ltd) |
| 728741000033117 | Hydromorphone 2.6mg capsules |
| 140841000033118 | Benylin with Codeine oral solution (Pfizer Consumer Healthcare Ltd) |
| 940241000033115 | MST Continus Suspension 200mg granules sachets (Napp Pharmaceuticals Ltd) |
| 3909741000033117 | Mabron 200mg modified-release tablets (Teva UK Ltd) |
| 4028641000033118 | Larapam SR 200mg tablets (Sandoz Ltd) |
| 4459541000033117 | Tramulief SR 200mg tablets (Advanz Pharma) |
| 12346741000033116 | Invodol SR 200mg tablets (Ennogen Healthcare Ltd) |
| 1462541000033112 | Tramadol 200mg modified-release tablets |
| 4817241000033113 | Filnarine SR 200mg tablets (Teva UK Ltd) |
| 941341000033117 | MST Continus 200mg tablets (Napp Pharmaceuticals Ltd) |
| 1549441000033115 | Zamadol SR 200mg capsules (Mylan) |
| 896441000033119 | Meptid 200mg tablets (Almirall Ltd) |
| 1561341000033119 | Zomorph 200mg modified-release capsules (Ethypharm UK Ltd) |
| 896341000033113 | Meptazinol 200mg tablets |
| 933141000033115 | Morphine 200mg modified-release tablets |
| 944241000033113 | MXL 200mg capsules (Napp Pharmaceuticals Ltd) |
| 3996541000033115 | Tradorec XL 200mg tablets (Endo Ventures Ltd) |
| 6133441000033110 | Tapentadol 200mg modified-release tablets |
| 1564741000033116 | Zydol SR 200mg tablets (Grunenthal Ltd) |
| 6134141000033116 | Palexia SR 200mg tablets (Grunenthal Ltd) |
| 11568441000033118 | Maneo 200mg modified-release tablets (Mylan) |
| 2078541000033112 | Dromadol SR 200mg tablets (Teva UK Ltd) |
| 1850241000033111 | Zydol XL 200mg tablets (Grunenthal Ltd) |
| 13712041000033119 | Brimisol PR 200mg tablets (Bristol Laboratories Ltd) |
| 4899441000033116 | Maxitram SR 200mg capsules (Chiesi Ltd) |
| 4824441000033111 | Marol 200mg modified-release tablets (Teva UK Ltd) |
| 1462841000033114 | Tramadol 200mg modified-release capsules |
| 1924341000033117 | Morphine 200mg modified-release capsules |
| 4417541000033116 | Zeridame SR 200mg tablets (Actavis UK Ltd) |
| 930141000033114 | Morphine 200mg modified-release granules sachets sugar free |
| 12350241000033114 | Tilodol SR 200mg tablets (Sandoz Ltd) |
| 12353841000033119 | Zytram SR 200mg tablets (Qdem Pharmaceuticals Ltd) |
| 4523341000033119 | Tramquel SR 200mg capsules (Mylan) |
| 2183541000033115 | Dromadol XL 200mg tablets (IVAX Pharmaceuticals UK Ltd) |
| 3344241000033112 | Zamadol 24hr 200mg modified-release tablets (Mylan) |
| 9061741000033112 | Shortec 20mg capsules (Qdem Pharmaceuticals Ltd) |
| 12664741000033110 | Oxypro 20mg modified-release tablets (Ridge Pharma Ltd) |
| 12666541000033114 | Ixyldone 20mg modified-release tablets (Morningside Healthcare Ltd) |
| 8048241000033115 | Oxylan 20mg modified-release tablets (Healthcare Pharma Ltd) |
| 1987741000033117 | OxyContin 20mg modified-release tablets (Napp Pharmaceuticals Ltd) |
| 9177241000033111 | Reltebon 20mg modified-release tablets (Accord Healthcare Ltd) |
| 1982041000033118 | Oxycodone 20mg modified-release tablets |
| 11808141000033112 | Leveraxo 20mg modified-release tablets (Mylan) |
| 940741000033114 | MST Continus Suspension 20mg granules sachets (Napp Pharmaceuticals Ltd) |
| 939541000033110 | Morphine 20mg tablets |
| 7886441000033118 | Longtec 20mg modified-release tablets (Qdem Pharmaceuticals Ltd) |
| 2068741000033113 | Morphine 20mg modified-release capsules |
| 934241000033112 | Morphine sulfate 20mg suppositories |
| 1987341000033118 | Oxycodone 20mg capsules |
| 10333441000033110 | Oxeltra 20mg modified-release tablets (Wockhardt UK Ltd) |
| 3032841000033119 | Morphine 20mg modified-release granules sachets sugar free |
| 930841000033115 | Morcap SR 20mg capsules (Hospira UK Ltd) |
| 336741000033117 | Codafen Continus tablets (Napp Pharmaceuticals Ltd) |
| 10985141000033118 | Carexil 20mg modified-release tablets (Sandoz Ltd) |
| 13708641000033113 | Oxycodone 20mg tablets |
| 8537541000033116 | Lynlor 20mg capsules (Accord Healthcare Ltd) |
| 11243341000033116 | Zomestine 20mg modified-release tablets (Accord Healthcare Ltd) |
| 12185541000033118 | Onexila XL 20mg tablets (Aspire Pharma Ltd) |
| 1278641000033115 | Sevredol 20mg tablets (Napp Pharmaceuticals Ltd) |
| 1988241000033112 | OxyNorm 20mg capsules (Napp Pharmaceuticals Ltd) |
| 10641741000033114 | Abtard 20mg modified-release tablets (Ethypharm UK Ltd) |
| 1273841000033110 | Sevredol Suppositories 20 mg |
| 12636941000033114 | Renocontin 20mg modified-release tablets (Glenmark Pharmaceuticals Europe Ltd) |
| 7859041000033111 | Dypracet 20mg/500mg tablets (Auden McKenzie (Pharma Division) Ltd) |
| 3229141000033111 | Ibuprofen 300mg modified-release / Codeine 20mg tablets |
| 3180241000033119 | Co-dydramol 20mg/500mg tablets |
| 1164541000033115 | Remedeine tablets (Crescent Pharma Ltd) |
| 5234541000033112 | Targinact 40mg/20mg modified-release tablets (Napp Pharmaceuticals Ltd) |
| 5234341000033117 | Oxycodone 40mg / Naloxone 20mg modified-release tablets |
| 1014941000033117 | Oramorph 20mg/ml concentrated oral solution (Boehringer Ingelheim Ltd) |
| 1744741000033112 | Morphine Sulfate Concentrated Oral Solution 20 mg/ml |
| 931841000033113 | Morphine sulfate 100mg/5ml oral solution unit dose vials sugar free |
| 1752041000033116 | Sevredol 20mg/ml concentrated oral solution (Napp Pharmaceuticals Ltd) |
| 931641000033112 | Morphine sulfate 20mg/ml oral solution sugar free |
| 9160141000033113 | Tapentadol 20mg/ml oral solution sugar free |
| 1014541000033111 | Oramorph 100mg/5ml oral solution unit dose vials (Boehringer Ingelheim Ltd) |
| 9160241000033118 | Palexia 20mg/ml oral solution (Grunenthal Ltd) |
| 738441000033110 | Hydromorphone 24mg modified-release capsules |
| 1036841000033111 | Palladone SR 24mg capsules (Napp Pharmaceuticals Ltd) |
| 6134241000033111 | Palexia SR 250mg tablets (Grunenthal Ltd) |
| 6133541000033111 | Tapentadol 250mg modified-release tablets |
| 609341000033114 | Fortral 25mg tablets (Zentiva) |
| 1066841000033118 | Pethidine Hydrochloride Tablets 25 mg |
| 1068041000033114 | Pentazocine 25mg tablets |
| 1036741000033118 | Palladone SR 2mg capsules (Napp Pharmaceuticals Ltd) |
| 11789141000033119 | Buprenorphine 2mg oral lyophilisates sugar free |
| 738341000033116 | Hydromorphone 2mg modified-release capsules |
| 11789341000033116 | Espranor 2mg oral lyophilisates (Martindale Pharmaceuticals Ltd) |
| 1014841000033113 | Oramorph 10mg/5ml oral solution (Boehringer Ingelheim Ltd) |
| 5891241000033112 | Co-dydramol 10mg/500mg/5ml oral solution |
| 1752541000033114 | Sevredol 10mg/5ml oral solution (Napp Pharmaceuticals Ltd) |
| 931741000033115 | Morphine sulfate 10mg/5ml oral solution unit dose vials sugar free |
| 13582941000033117 | Dihydrocodeine 10mg/5ml oral suspension |
| 442841000033115 | Dihydrocodeine 10mg/5ml oral solution |
| 3851441000033111 | Co-dydramol 10mg/500mg/5ml oral suspension |
| 1014441000033110 | Oramorph 10mg/5ml oral solution unit dose vials (Boehringer Ingelheim Ltd) |
| 10044141000033116 | Morphine 0.2% in Intrasite gel |
| 7859941000033112 | Morphine hydrochloride 10mg/5ml oral solution |
| 931541000033111 | Morphine sulfate 10mg/5ml oral solution |
| 3344341000033119 | Zamadol 24hr 300mg modified-release tablets (Mylan) |
| 3996641000033119 | Tradorec XL 300mg tablets (Endo Ventures Ltd) |
| 2183641000033119 | Dromadol XL 300mg tablets (IVAX Pharmaceuticals UK Ltd) |
| 1849941000033112 | Tramadol 300mg modified-release tablets |
| 1850341000033118 | Zydol XL 300mg tablets (Grunenthal Ltd) |
| 1025241000033119 | Oxycodone Pectinate Suppositories 30 mg |
| 934341000033119 | Morphine Hydrochloride Suppositories 30 mg |
| 295341000033111 | Co-Codamol 30/500 Caplets |
| 10641841000033116 | Abtard 30mg modified-release tablets (Ethypharm UK Ltd) |
| 6125141000033118 | Oxycodone 30mg modified-release tablets |
| 1014141000033119 | Oramorph Sr M/R tablets 30 mg |
| 371241000033116 | Codeine 30mg tablets |
| 941141000033115 | MST Continus 30mg tablets (Napp Pharmaceuticals Ltd) |
| 2753441000033117 | Filnarine SR 30mg tablets (Teva UK Ltd) |
| 6125541000033110 | OxyContin 30mg modified-release tablets (Napp Pharmaceuticals Ltd) |
| 9809741000033113 | Reltebon 30mg modified-release tablets (Accord Healthcare Ltd) |
| 468641000033111 | Dihydrocodeine Tartrate Tablets 30 mg |
| 944341000033115 | MXL 30mg capsules (Napp Pharmaceuticals Ltd) |
| 2912241000033112 | Morphgesic SR 30mg tablets (Advanz Pharma) |
| 10491641000033110 | Longtec 30mg modified-release tablets (Qdem Pharmaceuticals Ltd) |
| 1924041000033119 | Morphine 30mg modified-release capsules |
| 3032941000033110 | Morphine 30mg modified-release granules sachets sugar free |
| 12637241000033119 | Renocontin 30mg modified-release tablets (Glenmark Pharmaceuticals Europe Ltd) |
| 12664841000033117 | Oxypro 30mg modified-release tablets (Ridge Pharma Ltd) |
| 10333541000033111 | Oxeltra 30mg modified-release tablets (Wockhardt UK Ltd) |
| 940841000033116 | MST Continus Suspension 30mg granules sachets (Napp Pharmaceuticals Ltd) |
| 1561441000033113 | Zomorph 30mg modified-release capsules (Ethypharm UK Ltd) |
| 12666641000033110 | Ixyldone 30mg modified-release tablets (Morningside Healthcare Ltd) |
| 936641000033118 | Morphine 30mg modified-release tablets |
| 11808241000033117 | Leveraxo 30mg modified-release tablets (Mylan) |
| 1273941000033119 | Sevredol Suppositories 30 mg |
| 933941000033118 | Morphine sulfate 30mg suppositories |
| 468541000033110 | Dihydrocodeine 30mg tablets |
| 1698041000033118 | Solpadol 30mg/500mg capsules (Sanofi) |
| 12684741000033112 | Emcozin 30mg/500mg tablets (M & A Pharmachem Ltd) |
| 1352541000033110 | Solpadol 30mg/500mg effervescent tablets (Sanofi) |
| 295441000033117 | Co-codamol 30mg/500mg capsules |
| 1621841000033112 | Kapake Insts 30mg/500mg effervescent powder sachets (Galen Ltd) |
| 796641000033119 | Kapake 30mg/500mg tablets (Galen Ltd) |
| 1830841000033114 | Kapake 30mg/500mg capsules (Galen Ltd) |
| 1588741000033112 | Co-codamol 30mg/500mg effervescent powder sachets sugar free |
| 2746141000033115 | Zapain 30mg/500mg tablets (Advanz Pharma) |
| 3057541000033118 | Kapake 30mg/500mg effervescent tablets (Galen Ltd) |
| 1363841000033114 | Solpadol 30mg/500mg caplets (Sanofi) |
| 3180141000033114 | Co-dydramol 30mg/500mg tablets |
| 1479641000033111 | Tylex 30mg/500mg capsules (UCB Pharma Ltd) |
| 3331541000033110 | Medocodene 30mg/500mg effervescent tablets (Mylan) |
| 3334541000033113 | Medocodene 30mg/500mg capsules (UCB Pharma Ltd) |
| 1479741000033119 | Tylex 30mg/500mg effervescent tablets (UCB Pharma Ltd) |
| 2745941000033112 | Zapain 30mg/500mg capsules (Advanz Pharma) |
| 1157541000033117 | Remedeine Forte tablets (Crescent Pharma Ltd) |
| 7859141000033110 | Dypracet 30mg/500mg tablets (Auden McKenzie (Pharma Division) Ltd) |
| 326141000033118 | Co-codamol 30mg/500mg effervescent tablets |
| 370641000033114 | Co-codamol 30mg/500mg tablets |
| 468141000033118 | Distalgesic 32.5mg/325mg tablets (Meda Pharmaceuticals Ltd) |
| 373241000033115 | Co-proxamol 32.5mg/325mg tablets |
| 3180341000033112 | Tramadol 37.5mg / Paracetamol 325mg tablets |
| 5595941000033118 | Tramadol 37.5mg / Paracetamol 325mg effervescent tablets sugar free |
| 5596041000033111 | Tramacet 37.5mg/325mg effervescent tablets (Grunenthal Ltd) |
| 3057641000033117 | Tramacet 37.5mg/325mg tablets (Grunenthal Ltd) |
| 2645041000033118 | Codeine 15mg/5ml linctus sugar free |
| 335841000033117 | Codeine Phosphate Linctus 15 mg/5 ml |
| 368441000033116 | Codeine 15mg/5ml linctus |
| 831641000033117 | Linctus Of Codeine Linctus 15 mg/5 ml |
| 624441000033112 | Galcodine 15mg/5ml linctus (Thornton & Ross Ltd) |
| 335641000033118 | Codeine Linctus Diabetic Linctus 15 mg/5 ml |
| 1850441000033112 | Zydol XL 400mg tablets (Grunenthal Ltd) |
| 2183741000033111 | Dromadol XL 400mg tablets (IVAX Pharmaceuticals UK Ltd) |
| 1850041000033115 | Tramadol 400mg modified-release tablets |
| 3344441000033113 | Zamadol 24hr 400mg modified-release tablets (Mylan) |
| 1987841000033110 | OxyContin 40mg modified-release tablets (Napp Pharmaceuticals Ltd) |
| 433141000033118 | DF 118 Forte 40mg tablets (Martindale Pharmaceuticals Ltd) |
| 9177141000033116 | Reltebon 40mg modified-release tablets (Accord Healthcare Ltd) |
| 11808341000033110 | Leveraxo 40mg modified-release tablets (Mylan) |
| 462841000033114 | Dihydrocodeine 40mg tablets |
| 11243441000033110 | Zomestine 40mg modified-release tablets (Accord Healthcare Ltd) |
| 12666741000033118 | Ixyldone 40mg modified-release tablets (Morningside Healthcare Ltd) |
| 7886541000033117 | Longtec 40mg modified-release tablets (Qdem Pharmaceuticals Ltd) |
| 12185641000033117 | Onexila XL 40mg tablets (Aspire Pharma Ltd) |
| 10333641000033112 | Oxeltra 40mg modified-release tablets (Wockhardt UK Ltd) |
| 8048341000033113 | Oxylan 40mg modified-release tablets (Healthcare Pharma Ltd) |
| 12664941000033113 | Oxypro 40mg modified-release tablets (Ridge Pharma Ltd) |
| 1982141000033119 | Oxycodone 40mg modified-release tablets |
| 12637341000033112 | Renocontin 40mg modified-release tablets (Glenmark Pharmaceuticals Europe Ltd) |
| 10641941000033112 | Abtard 40mg modified-release tablets (Ethypharm UK Ltd) |
| 1036941000033115 | Palladone SR 4mg capsules (Napp Pharmaceuticals Ltd) |
| 738541000033111 | Hydromorphone 4mg modified-release capsules |
| 2068841000033115 | Morphine 50mg modified-release capsules |
| 4523041000033116 | Tramquel SR 50mg capsules (Mylan) |
| 1702241000033118 | Tramadol 50mg effervescent powder sachets sugar free |
| 1564341000033117 | Zydol 50mg capsules (Grunenthal Ltd) |
| 1276641000033119 | Sevredol 50mg tablets (Napp Pharmaceuticals Ltd) |
| 13300641000033110 | Pethidine 50mg capsules |
| 1063141000033116 | Pentazocine 50mg suppositories |
| 1454241000033114 | Tramadol 50mg capsules |
| 2980441000033112 | Zamadol Melt 50mg tablets (Mylan) |
| 6132041000033110 | Tapentadol 50mg tablets |
| 607341000033118 | Fortral 50mg suppositories (Sterwin Medicines) |
| 1065641000033110 | Pethidine 50mg tablets |
| 2980341000033118 | Tramadol 50mg orodispersible tablets sugar free |
| 1454541000033111 | Tramake 50mg capsules (Galen Ltd) |
| 2180441000033114 | Morphine Sulfate Suppositories 50 mg |
| 4259241000033118 | Zydol SR 50mg tablets (Grunenthal Ltd) |
| 938241000033117 | Morphine 50mg tablets |
| 1564841000033114 | Zydol 50mg soluble tablets (Grunenthal Ltd) |
| 6132241000033119 | Palexia 50mg tablets (Grunenthal Ltd) |
| 930941000033111 | Morcap SR 50mg capsules (Hospira UK Ltd) |
| 1549541000033119 | Zamadol SR 50mg capsules (Mylan) |
| 599141000033118 | Fortral Capsules 50 mg |
| 6133841000033113 | Palexia SR 50mg tablets (Grunenthal Ltd) |
| 1548041000033116 | Zamadol 50mg capsules (Mylan) |
| 1702041000033114 | Tramake Insts 50mg sachets (Galen Ltd) |
| 1044641000033117 | Pentazocine 50mg capsules |
| 4259141000033113 | Tramadol 50mg modified-release tablets |
| 4899141000033112 | Maxitram SR 50mg capsules (Chiesi Ltd) |
| 1465741000033113 | Tramadol 50mg soluble tablets sugar free |
| 6133141000033119 | Tapentadol 50mg modified-release tablets |
| 1462941000033118 | Tramadol 50mg modified-release capsules |
| 8048041000033111 | Oxylan 5mg modified-release tablets (Healthcare Pharma Ltd) |
| 13708741000033116 | Oxyact 5mg tablets (Kent Pharmaceuticals Ltd) |
| 2748441000033110 | Oxycodone 5mg modified-release tablets |
| 11808441000033116 | Leveraxo 5mg modified-release tablets (Mylan) |
| 430541000033115 | Dextromoramide 5mg tablets |
| 10333141000033119 | Oxeltra 5mg modified-release tablets (Wockhardt UK Ltd) |
| 12636441000033116 | Renocontin 5mg modified-release tablets (Glenmark Pharmaceuticals Europe Ltd) |
| 2748541000033111 | OxyContin 5mg modified-release tablets (Napp Pharmaceuticals Ltd) |
| 7886241000033119 | Longtec 5mg modified-release tablets (Qdem Pharmaceuticals Ltd) |
| 12666841000033111 | Ixyldone 5mg modified-release tablets (Morningside Healthcare Ltd) |
| 1987141000033116 | Oxycodone 5mg capsules |
| 12665041000033113 | Oxypro 5mg modified-release tablets (Ridge Pharma Ltd) |
| 13708441000033111 | Oxycodone 5mg tablets |
| 1041341000033111 | Palfium 5mg tablets (Roche Products Ltd) |
| 8537341000033111 | Lynlor 5mg capsules (Accord Healthcare Ltd) |
| 9177041000033115 | Reltebon 5mg modified-release tablets (Accord Healthcare Ltd) |
| 1988041000033116 | OxyNorm 5mg capsules (Napp Pharmaceuticals Ltd) |
| 940441000033119 | MST Continus 5mg tablets (Napp Pharmaceuticals Ltd) |
| 11243141000033119 | Zomestine 5mg modified-release tablets (Accord Healthcare Ltd) |
| 10984841000033113 | Carexil 5mg modified-release tablets (Sandoz Ltd) |
| 930341000033112 | Morphine 5mg modified-release tablets |
| 10641441000033119 | Abtard 5mg modified-release tablets (Ethypharm UK Ltd) |
| 9061541000033116 | Shortec 5mg capsules (Qdem Pharmaceuticals Ltd) |
| 4898141000033118 | Oxycodone 10mg / Naloxone 5mg modified-release tablets |
| 4898441000033114 | Targinact 10mg/5mg modified-release tablets (Napp Pharmaceuticals Ltd) |
| 13576541000033116 | Myloxifin 10mg/5mg modified-release tablets (Zentiva) |
| 368541000033115 | Codeine 25mg/5ml oral solution |
| 2972441000033117 | Co-proxamol 32.5mg/325mg/5ml oral suspension |
| 944441000033114 | MXL 60mg capsules (Napp Pharmaceuticals Ltd) |
| 9809841000033115 | Reltebon 60mg modified-release tablets (Accord Healthcare Ltd) |
| 10492041000033114 | Longtec 60mg modified-release tablets (Qdem Pharmaceuticals Ltd) |
| 10642041000033118 | Abtard 60mg modified-release tablets (Ethypharm UK Ltd) |
| 940341000033113 | MST Continus Suspension 60mg granules sachets (Napp Pharmaceuticals Ltd) |
| 1561541000033114 | Zomorph 60mg modified-release capsules (Ethypharm UK Ltd) |
| 2912341000033119 | Morphgesic SR 60mg tablets (Advanz Pharma) |
| 941241000033110 | MST Continus 60mg tablets (Napp Pharmaceuticals Ltd) |
| 6125641000033111 | OxyContin 60mg modified-release tablets (Napp Pharmaceuticals Ltd) |
| 1924141000033115 | Morphine 60mg modified-release capsules |
| 930241000033119 | Morphine 60mg modified-release granules sachets sugar free |
| 936741000033110 | Morphine 60mg modified-release tablets |
| 11808541000033115 | Leveraxo 60mg modified-release tablets (Mylan) |
| 468741000033119 | Dihydrocodeine 60mg modified-release tablets |
| 416141000033118 | Dextropropoxyphene 60mg capsules |
| 371341000033114 | Codeine 60mg tablets |
| 6125241000033113 | Oxycodone 60mg modified-release tablets |
| 2753541000033116 | Filnarine SR 60mg tablets (Teva UK Ltd) |
| 12637441000033118 | Renocontin 60mg modified-release tablets (Glenmark Pharmaceuticals Europe Ltd) |
| 1014241000033114 | Oramorph Sr M/R tablets 60 mg |
| 10333741000033115 | Oxeltra 60mg modified-release tablets (Wockhardt UK Ltd) |
| 433341000033115 | DHC Continus 60mg tablets (Napp Pharmaceuticals Ltd) |
| 12665141000033112 | Oxypro 60mg modified-release tablets (Ridge Pharma Ltd) |
| 472441000033119 | Doloxene Capsules 65 mg |
| 13417741000033113 | Co-codamol 30mg/500mg/5ml oral solution sugar free |
| 931941000033117 | Morphine sulfate 30mg/5ml oral solution unit dose vials sugar free |
| 1014641000033112 | Oramorph 30mg/5ml oral solution unit dose vials (Boehringer Ingelheim Ltd) |
| 1043041000033113 | Paramol tablets (SSL International Plc) |
| 86841000033118 | Aspav dispersible tablets (Actavis UK Ltd) |
| 3849841000033110 | Aspirin 500mg / Papaveretum 7.71mg dispersible tablets sugar free |
| 12471541000033111 | Tramadol 75mg / Dexketoprofen 25mg tablets |
| 6132341000033112 | Palexia 75mg tablets (Grunenthal Ltd) |
| 2078141000033115 | Tramadol 75mg modified-release tablets |
| 6132141000033114 | Tapentadol 75mg tablets |
| 2078241000033110 | Dromadol SR 75mg tablets (IVAX Pharmaceuticals UK Ltd) |
| 12353941000033110 | Zytram SR 75mg tablets (Qdem Pharmaceuticals Ltd) |
| 11783041000033115 | Tramadol 75mg / Paracetamol 650mg tablets |
| 932041000033111 | Morphine Oral solution 8.4 mg/ml |
| 11243541000033111 | Zomestine 80mg modified-release tablets (Accord Healthcare Ltd) |
| 10642141000033119 | Abtard 80mg modified-release tablets (Ethypharm UK Ltd) |
| 7886641000033116 | Longtec 80mg modified-release tablets (Qdem Pharmaceuticals Ltd) |
| 12185741000033114 | Onexila XL 80mg tablets (Aspire Pharma Ltd) |
| 12665241000033117 | Oxypro 80mg modified-release tablets (Ridge Pharma Ltd) |
| 12667041000033119 | Ixyldone 80mg modified-release tablets (Morningside Healthcare Ltd) |
| 1987941000033119 | OxyContin 80mg modified-release tablets (Napp Pharmaceuticals Ltd) |
| 10333841000033113 | Oxeltra 80mg modified-release tablets (Wockhardt UK Ltd) |
| 11808641000033119 | Leveraxo 80mg modified-release tablets (Mylan) |
| 1982541000033111 | Oxycodone 80mg modified-release tablets |
| 9176841000033112 | Reltebon 80mg modified-release tablets (Accord Healthcare Ltd) |
| 8048441000033119 | Oxylan 80mg modified-release tablets (Healthcare Pharma Ltd) |
| 1365241000033111 | Solpadeine Tablets |
| 11789241000033114 | Buprenorphine 8mg oral lyophilisates sugar free |
| 3934441000033118 | Codis 500 dispersible tablets (Reckitt Benckiser Healthcare (UK) Ltd) |
| 738641000033112 | Hydromorphone 8mg modified-release capsules |
| 918241000033114 | Migraleve tablets (McNeil Products Ltd) |
| 318141000033113 | Codis Dispersible tablets |
| 1512141000033114 | Veganin tablets (Omega Pharma Ltd) |
| 916741000033116 | Migraleve Pink tablets (McNeil Products Ltd) |
| 86941000033114 | Aspirin Paracetamol And Codeine Tablets |
| 1037041000033119 | Palladone SR 8mg capsules (Napp Pharmaceuticals Ltd) |
| 1040041000033117 | Paracetamol & Codeine Tablets |
| 371041000033112 | Co-codaprin 8mg/400mg dispersible tablets |
| 4590641000033113 | Co-codamol 8mg/500mg caplets (Vantage) |
| 11789441000033110 | Espranor 8mg oral lyophilisates (Martindale Pharmaceuticals Ltd) |
| 8246141000033119 | Solpadeine Plus capsules (Omega Pharma Ltd) |
| 4432041000033115 | Paracodol 8mg/500mg capsules (Bayer Plc) |
| 1030141000033119 | Paracodol 8mg/500mg effervescent tablets (Bayer Plc) |
| 370941000033119 | Co-codaprin 8mg/400mg tablets |
| 3934341000033112 | Aspirin 500mg / Codeine 8mg dispersible tablets sugar free |
| 294841000033118 | Co-codamol 8mg/500mg capsules |
| 922541000033116 | Migraleve Yellow tablets (McNeil Products Ltd) |
| 372941000033118 | Co-codamol 8mg/500mg tablets |
| 11736241000033114 | Solpadeine Plus soluble tablets (Omega Pharma Ltd) |
| 373041000033111 | Co-codamol 8mg/500mg effervescent tablets |
| 13497541000033118 | Co-codamol 8mg/500mg effervescent tablets sugar free |
| 433541000033110 | DHC Continus 90mg tablets (Napp Pharmaceuticals Ltd) |
| 944541000033110 | MXL 90mg capsules (Napp Pharmaceuticals Ltd) |
| 469541000033115 | Dihydrocodeine 90mg modified-release tablets |
| 2068441000033118 | Morphine 90mg modified-release capsules |
