# Supplementary material for: Trends of long-term opioid therapy and subsequent discontinuation among people with chronic non-cancer pain in UK primary care: A retrospective cohort study
Source: PLoS One. 2025 Jun 26;20(6):e0326604. doi: 10.1371/journal.pone.0326604 (PMC12200650; doi:10.1371/journal.pone.0326604)
Supplement: S4 Table — (DOCX) [file pone.0326604.s007.docx]

# **S4 Table. Results of structural break analysis using annual number of opioid users between 2000-2020**

No additional breaks found for 4 breaks.
Sequential test for multiple breaks at unknown breakpoints
(Ditzen, Karavias & Westerlund. 2021)

----------------- Bai & Perron Critical Values -----------------

Test 1% Critical 5% Critical 10% Critical
 Statistic Value Value Value

| F (1\|0) | 15.47 | 12.29 | 8.58 | 7.04 |
| --- | --- | --- | --- | --- |
| F (2\|1) | 83.65 | 13.89 | 10.13 | 8.51 |
| F (3\|2) | 27.26 | 14.80 | 11.14 | 9.41 |

Detected number of breaks: 3 3 3

| The detected number of breaks indicates the highest number of breaks for which the null hypothesis is rejected. |
| --- |
| Estimation of break points |
| T=10 |
| SSR=9.98e+07 |
| Trimming=0.15 |
|  |

| # | Index | Date | 95% Confidence Interval |
| --- | --- | --- | --- |
| 1 | 4 | 2014 | 2013 to 2015 |
| 2 | 8 | 2018 | 2017 to 2019 |
| 3 | 9 | 2019 | 2018 to 2020 |
