# Supplementary material for: Trends of long-term opioid therapy and subsequent discontinuation among people with chronic non-cancer pain in UK primary care: A retrospective cohort study
Source: PLoS One. 2025 Jun 26;20(6):e0326604. doi: 10.1371/journal.pone.0326604 (PMC12200650; doi:10.1371/journal.pone.0326604)
Supplement: S5 Table — (DOCX) [file pone.0326604.s008.docx]

# **S5 Table. Incidence rate ratios of negative binominal regression models for yearly rates of incident opioid users, L-TOT users and L-TOT discontinuers from 2009 to 2019**

|  | Incident opioid user  (n=2,839,161) | L-TOT users  (n=324,877) | L-TOT Discontinuers  (n=15,484) |
| --- | --- | --- | --- |
| **Regression model** | **IRR, 95%CI** | **IRR, 95%CI** | **IRR, 95%CI** |
| Time (β1) | 0.992 (0.981, 1.003) | 0.978 (0.974, 0.982) * | 0.980 (0.969, 0.992) * |
| Indicator (β2) | 1.030 (0.973, 1.089) | 1.008 (0.989, 1.028) | 1.024 (0.962, 1.089) |
| Interaction term (β3) | 0.922 (0.905, 0.939) * | 1.020 (1.013, 1.027) * | 0.981 (0.960, 1.003) |
