# Supplementary material for: Trends of long-term opioid therapy and subsequent discontinuation among people with chronic non-cancer pain in UK primary care: A retrospective cohort study
Source: PLoS One. 2025 Jun 26;20(6):e0326604. doi: 10.1371/journal.pone.0326604 (PMC12200650; doi:10.1371/journal.pone.0326604)
Supplement: S6 Table — (DOCX) [file pone.0326604.s009.docx]

# **S6 Table. Incidence rate ratios of Poisson regression models for yearly rates of incident opioid users, L-TOT users and L-TOT discontinuers during 2009-2013 and 2015-2019**

|  | Incident opioid user  (n=2,558,642) | L-TOT users  (n=293,128) | L-TOT Discontinuers  (n=14,014) |
| --- | --- | --- | --- |
| **Regression model** | **IRR, 95%CI** | **IRR, 95%CI** | **IRR, 95%CI** |
| Time (β1) | 1.001 (0.988, 1.014) | 0.974 (0.971, 0.978) * | 0.987 (0.972, 1.003) |
| Indicator (β2) | 0.988 (0.928, 1.052) | 1.026 (1.009, 1.044) * | 0.990 (0.916, 1.070) |
| Interaction term (β3) | 0.913 (0.897, 0.930) * | 1.024 (1.019, 1.029) * | 0.974 (0.951, 0.998) * |
